# Biglycan Interacts with Type I Insulin-like Receptor (IGF-IR) Signaling Pathway to Regulate Osteosarcoma Cell Growth and Response to Chemotherapy

**DOI:** 10.3390/cancers14051196

**Published:** 2022-02-25

**Authors:** Eirini-Maria Giatagana, Aikaterini Berdiaki, Margrethe Gaardløs, Sergey A. Samsonov, George N. Tzanakakis, Dragana Nikitovic

**Affiliations:** 1 Laboratory of Histology-Embryology, Medical School, University of Crete, 71003 Heraklion, Greece; eirini_gt@hotmail.com (E.-M.G.); berdiaki@uoc.gr (A.B.); tzanakak@uoc.gr (G.N.T.); 2 Department of Theoretical Chemistry, Faculty of Chemistry, University of Gdańsk, ul. Wita Stwosza 63, 80-308 Gdansk, Poland; margrethe.gaardlos@gmail.com (M.G.); sergey.samsonov@ug.edu.pl (S.A.S.)

**Keywords:** biglycan, insulin-like growth factor receptor I, extracellular matrix, osteosarcoma, chemoresistance

## Abstract

**Simple Summary:**

Osteosarcoma (OS) is an aggressive, primary bone cancer. OS cells produce altered osteoid whose components participate in signaling correlated to the development of this cancer. Biglycan (BGN), a proteoglycan, is correlated to aggressive OS type and resistance to chemotherapy. A constitutive signaling of insulin-like growth factor receptor I (IGF-IR) signaling in sarcoma progression was established. We showed that biglycan binds IGF-IR resulting in prolonged IGF-IR activation, nuclear translocation, and growth response of the poorly-differentiated MG63 cells correlated to increased aggressiveness markers expression and enhanced chemoresistance. This mechanism is not valid in moderately and well-differentiated, biglycan non-expressing U-2OS and Saos-2 OS cells.

**Abstract:**

Osteosarcoma (OS) is a mesenchymally derived, aggressive bone cancer. OS cells produce an aberrant nonmineralized or partly mineralized extracellular matrix (ECM) whose components participate in signaling pathways connected to specific pathogenic phenotypes of this bone cancer. The expression of biglycan (BGN), a secreted small leucine-rich proteoglycan (SLRP), is correlated to aggressive OS phenotype and resistance to chemotherapy. A constitutive signaling of IGF-IR signaling input in sarcoma progression has been established. Here, we show that biglycan activates the IGF-IR signaling pathway to promote MG63 biglycan-secreting OS cell growth by forming a complex with the receptor. Computational models of IGF-IR and biglycan docking suggest that biglycan binds IGF-IR dimer via its concave surface. Our binding free energy calculations indicate the formation of a stable complex. Biglycan binding results in prolonged IGF-IR activation leading to protracted IGF-IR-dependent cell growth response of the poorly-differentiated MG63 cells. Moreover, biglycan facilitates the internalization (*p* ≤ 0.01, *p* ≤ 0.001) and sumoylation-enhanced nuclear translocation of IGF-IR (*p* ≤ 0.05) and its DNA binding in MG63 cells (*p* ≤ 0.001). The tyrosine kinase activity of the receptor mediates this mechanism. Furthermore, biglycan downregulates the expression of the tumor-suppressor gene, PTEN (*p* ≤ 0.01), and increases the expression of endothelial–mesenchymal transition (EMT) and aggressiveness markers vimentin (*p* ≤ 0.01) and fibronectin (*p* ≤ 0.01) in MG63 cells. Interestingly, this mechanism is not valid in moderately and well-differentiated, biglycan non-expressing U-2OS and Saos-2 OS cells. Furthermore, biglycan exhibits protective effects against the chemotherapeutic drug, doxorubicin, in MG63 OS cells (*p* ≤ 0.01). In conclusion, these data indicate a potential direct and adjunct therapeutical role of biglycan in osteosarcoma.

## 1. Introduction

Osteosarcoma (OS), a highly malignant neoplasm, is the most common primary tumor of the bone. This cancer primarily affects children and adolescents between 10 and 20 years but shows a second peak of incidence in the older population [1,2]. Bone cells typically form osteoid, a highly specialized organic mineralized extracellular matrix (ECM) mainly consisting of type I collagen, glycoproteins, and proteoglycans (PGs). Notably, the OS cells produce an aberrant nonmineralized or partly mineralized ECM [3], whose components participate in signaling pathways connected to specific pathogenic phenotypes of this bone cancer [4,5].

PGs are a family of proteins that undergo post-translational modifications, as their protein core is covalently linked with one or more glycosaminoglycan (GAG) chains [6]. The small leucine-rich proteoglycans (SLRPs) are a distinct family of PGs with unique characteristics. They consist of a small protein core (36–42 kDa) with several leucine-rich repeats (LRRs) and are substituted with a various number of GAG chains [7,8]. Their classification into five distinct classes is based on the conservation of the amino acid residues of the protein core, the organization of disulfide bonds at the N- and C-terminal regions, and their gene homology [9]. Many studies have shown that SLRPs significantly contribute to matrix organization and participate in cell communication with their microenvironment [10]. SLRPs undergo an astonishing range of protein–protein interactions, including binding to growth factors, cell surface receptors, and collagens [7,9]. Indeed, by regulating signal transduction mechanisms, these molecules affect basal cellular functions, such as proliferation, migration, and differentiation [8,11]. An important example is that SLRP interaction with growth factors, and their respective tyrosine kinase receptors, regulates key downstream intracellular signaling pathways [12,13,14].

Biglycan is a class I SLRP member glycosylated by two chondroitin or dermatan sulfate side chains, covalently linked to the N-terminal of its protein core [15]. The biglycan monomer is arch-shaped, consisting of a right-handed spiral of 12 leucine-rich repeats. Each repeat is a short β-strand linked by loops, strands, and short helical segments [16]. This SLRP is initially synthesized as a precursor containing an N-terminal propeptide which is shed through the activity of bone morphogenetic protein 1 (BMP 1) to form the mature form [17]. Secreted biglycan interacts via its protein core or GAG chains with different ECM constituents, which results in its sequestration [18]. Unsequestered biglycan binds through its concave surface and modulates the activity of cytokines and growth factors, including Wnt-1-induced, secreted protein 1 (WISP1) [19], BMP-2 and -4 [20,21], transforming growth factor-beta (TGF-β) [22], and tumor necrosis factor (TNF)-α [23]. Biglycan was identified to be associated with the process of carcinogenesis [24], although its role in tumorigenesis is not fully established.

Insulin-like growth factor I (IGF-I) is a well-established anabolic growth factor with proven oncogenic properties [25]. Insulin-like growth factor receptor IGF-IR mediates a variety of its actions [11]. The IGF-IR is a dimeric (heterotetrameric) transmembrane glycoprotein with a similar structure to the insulin receptor (IR). The extracellular region of the mature protein is involved in ligand binding, and the intracellular region encompasses the tyrosine kinase domain [26,27]. Upon ligand binding, the IGF-IR functions are performed through two main signaling pathways, PI3K (phosphoinositide 3-kinase/serine/threonine kinase)/Akt and MAPK (mitogen-activated protein kinase)/ERK (extracellular-signal-regulated kinase) [28]. IGF-I/IGF-IR signaling axis is strongly associated with mesenchymal cancers [13,29,30]. Indeed, a poor prognosis of osteosarcoma patients expressing IGF-I was determined by implementing tissue microarray analysis [31], and a generalized IGF-IR signaling input in sarcoma progression was demonstrated by meta-analysis correlating IGF-IR expression with poor outcomes in sarcoma patients [32]. More recent studies correlate the hyperactive receptor with the development of therapy resistance in many cancers [33,34,35]. Moreover, inhibiting IGF-IR tyrosine kinase activity enhances an IGF-IR/β-arrestin-1/ERK signaling axis, resulting in tumor resistance to this therapy [36].

Some reports relate biglycan function with the pathogenesis of osteosarcoma. Indeed, biglycan interacts with the IGF-IR signaling pathway to enhance the proliferation of osteosarcoma cells [30]. This could be clinically important as increased IGF-I expression, and IGF-IR activity are evident in osteosarcoma [37,38]. Moreover, Mintz et al. have shown a significant correlation of biglycan expression with chemoresistant pediatric osteosarcomas [39]. Considering the synergistic action of biglycan with the IGF-IR signaling cascade in controlling MG63 osteosarcoma cell growth, we examined the putative mechanisms involved in their co-action effect. Our results demonstrate that biglycan forms a complex with the activated IGF-IR and controls its translocation to the nucleus. This signaling results in increased MG63 osteosarcoma cells’ aggressiveness and chemoresistance to doxorubicin, suggesting a potential direct and adjunct therapeutical implication of biglycan in osteosarcoma.

## 2. Materials and Methods

### 2.1. Materials

Recombinant human biglycan (AR50812PU-S 1 mg/mL) was obtained from OriGene, Rockville, USA. A specific inhibitor of IGF-IR (AG1024 121767, Calbiochem, San Diego, CA, USA) was used. Doxorubicin hydrochloride (D1515-10MG) was purchased from Sigma-Aldrich, St Luis, MO, USA. Primary antibodies from Santa Cruz Biotechnology Inc were used, including anti-IGF-IR (sc-81464; mouse monoclonal; 1/100 dilution), anti-pIGF-IR (sc-135767; mouse monoclonal; 1/100 dilution for Western blots and 1/50 for immunofluoresence), anti-biglycan (sc-100857; mouse monoclonal; 1/100 dilution), anti-SUMO-1 (sc-5308; mouse monoclonal; 1/100 dilution), anti-tubulin (sc-5286; mouse monoclonal; 1/100 dilution), anti-lamin B1 (sc-374015; mouse monoclonal; 1/100 dilution), anti-vimentin antibody (sc-6260; mouse monoclonal; 1/200 dilution), and anti-fibronectin antibody (sc-59826; mouse monoclonal; 1/200 dilution). Protein A/G PLUS-Agarose (sc-2003) was also obtained from Santa Cruz Biotechnology, Inc. (Dallas, TX, USA). Anti-actin (MAB1501; mouse monoclonal; 1/5.000 dilution), and the secondary-HRP anti-mouse antibody (AP192P; 1/5.000 dilution) were purchased by EMD Millipore, Burlington, VT, USA. Anti-biglycan antibody from Elabscience (Houston, TX, USA) was also used for immunofluorescence experiments (E-AB-11001; rabbit polyclonal; 1/200 dilution). From Thermofisher Scientific (Waltham, MA, USA) were obtained anti-mouse Alexa Fluor 488 (A21202; 1/200 dilution), anti-mouse Alexa Fluor 555 (A21422; 1/200 dilution), and anti-rabbit Alexa Fluor 488 (A21206; 1/200 dilution) antibodies and TO-PRO-3 iodide (T3605; 1/300) for immunofluorescence experiments.

#### 2.1.1. Cells and Cell Culture

MG63 (ATCC^®^ CRL1427™), Saos-2 (ATCC^®^ HTB-85™), and U-2OS (ATCC^®^ HTB-96™) human osteosarcoma cell lines were utilized. Cells were grown in DMEM (Gibco-41966-029) supplemented with 10% fetal bovine serum (FBS; Invitrogen 10500-064; heat-inactivated), gentamycin (Invitrogen, Waltham, MA, USA; 15710-049) and penicillin/streptomycin (100 units/mL; Biosera LMA4118). Cells were cultured at 37 °C and 5% CO_2_ conditions.

#### 2.1.2. Transfection with siRNA

For transfection experiments, siRNAs specific for biglycan (sibgn; stealth siRNAs HSS184531; Invitrogen; Thermo Fisher Scientific, Inc., Waltham, MA, USA) and RNAi negative control (si-scr; medium GC content negative control; Invitrogen; Thermo Fisher Scientific, Inc.) and serum- and antibiotic-free medium were used. For transfection, siRNA, and Lipofectamine 2000 (11668 027; Invitrogen; Thermo Fisher Scientific, Inc.) were diluted in Opti-MEM I Reduced Serum Medium (31985-070; Invitrogen; Thermo Fisher Scientific, Inc.). Following 5 min of incubation at room temperature, diluted Lipofectamine 2000 was mixed with diluted siRNA (100 nm) for 20 min at room temperature to allow siRNA-liposome complexes to form and was added to cell layers. Transfection was allowed to take place during 24 h when the medium was replaced with fresh (10% FBS) containing antibiotics, and the incubation period continued for 48 h. Doxorubicin treatment (0.1 μg/mL) was also added for the next 48 h at 37 °C and 5% CO_2_ in 10% FBS medium. Cells were then harvested, and mRNA was extracted. All transfection experiments were repeated at least 3 times and performed in triplicate.

#### 2.1.3. Proliferation Assay

Growing cells from confluent cultures were seeded in black 96-well plates at a density of 1500 cells/well (MG63), 4000 cells/well (U-2OS), and 20,000 cells/well (Saos-2) in 200 µL of DMEM. The cell density number was selected from optimization experiments (Figure S1). The cells were allowed to rest overnight. If necessary, transfection with short interfering RNAs (siRNAs) was performed in a serum-free medium without antibiotics for 24 h and then replaced with fresh medium (10% FBS) with antibiotics, with or without doxorubicin treatment (0.1 μg/mL). Biglycan treatment was added in 0% FBS medium for the next 48 h at 37 °C and 5% CO_2_. Biglycan concentration, used in the experiments, was selected from optimization experiments (Figure S2). The cells were then lysed, and their number was calculated using the CyQUANT fluorometric assay (C7026; Thermo Fisher Scientific, Inc.) according to the manufacturer’s instructions. Fluorescence was measured in a Fluorometer (BioTek/ Agilent Instruments, Inc, Santa Clara, CA, USA.) using the proposed excitation (485 nm) and emission filters (528 nm). A separate standard curve was used to convert fluorescence units to cell numbers. All experiments were performed in triplicate.

#### 2.1.4. RNA Isolation and Reverse Transcription Quantitative PCR (RT qPCR)

According to the manufacturer’s instructions, total ribonucleic acid isolation was performed using TRIzol (15596026; Invitrogen; Thermo Fisher Scientific, Inc.). Total RNA (1 µg) was added for cDNA synthesis using the Takara (RR037A) RT cDNA synthesis kit. For semi quantification of the genes of interest, qPCR reactions were performed on a Mx300P cycler using the Universal qPCR kit (KK4602; KAPA Biosystems-Roche, Basel, Switzerland) in a total volume of 20 µL. The thermocycling conditions were as follows: 94 °C for 15 min, 40 cycles at 94 °C for 20 s, 55 °C for 30 s, 72 °C for 30 s, 72 °C for 10 min. The PCR primer sequences were as follows: GAPDH forward, 5′-GGAAGGTGAAGGTCGGAGTCA-3′ and reverse, 5′-GTC ATTGATGGCAACAATATCCACT-3′; biglycan forward, 5′-TCTGAAGTCTGTGCCCAA-3′ and reverse, 5′-TCTGAGATGCGCAGGTA-3′; PTEN forward, 5′-CCAGTGGCACTGTTGTTTCACA -3′ and reverse, 5′-CAGGTAACGGCTGAGGGAGCTC -3′. Standard curves were run in each optimized assay, which produced a linear plot of the threshold cycle Ct (dRn) against the initial quantity (copies). The amount of each target was semi quantified based on the concentration of the standard curve and was presented as arbitrary units. GAPDH was utilized as a housekeeping gene.

#### 2.1.5. Protein Immunoprecipitation

For immunoprecipitation with protein A/G and agarose, after treatments, cells were detached and diluted in 1 mL RIRAsolution (50 mMTris-HCl, 1% NP-40, 0.25% Na-Deoxycholate, 150 mM NaCl, 1 mM EDTA with protease and phosphatase inhibitors). One hundred microliters from this dilution was frozen at −80 °C. In the rest, 900 μL of every protein sample, 30 μL of the primary antibody was added, and tubes were incubated on a rotating platform overnight at 4 °C (3 μg of primary antibody for 1 mg of total protein). The next day, 30 μL of protein A/G with agarose (SantaCruz) was added for 4 h at 4 °C, and tubes were incubated again on a rotating platform. After centrifugation at 1000 rpm for 1 min (4 °C), the precipitate was diluted again with 1 mL RIPA solution. This process was repeated twice more. Finally, the precipitate was diluted in 30 μL 2x dye, and the samples were utilized for Western blot analysis. All the immunoprecipitation experiments were conducted with primary antibodies against proteins whose expression is not affected by biglycan treatment. We tested the total protein expression of primary antibodies in Western blots to normalize the results of the immunoprecipitated protein levels. The technique of protein immunoprecipitation was optimized, utilizing a known complex as a positive control. Beads without antibody binding or detected with an antibody against GAPDH, which is not related to the target protein, were used as negative controls and are deposited in Supplementary Figure S3.

#### 2.1.6. Nuclear and Cytoplasmic Extract Separation

Treated cells in T25 flasks were detached using trypsin-EDTA (Biosera, Shanghai, China; LMT1706), deactivated with PBS, and centrifuged at 1.100 rpm for 5 min. Supernatants were discarded, and the pellets were resuspended in 250 µL of ice-cold PBS supplemented with protease and phosphatase inhibitors. After centrifugation at 1.100 rpm for 5 min at 4 °C the pellets were resuspended in 200 µL of 5x CPV NP-40 lysis buffer (10 mMTris-HCl, 10 mM NaCl, 3 mM MgCl_2_, 0.5% NP-40 with protease and phosphatase inhibitors). Tubes were incubated on a rotating platform at 4 °C for 10 min and centrifuged strictly at 1.000 rpm for 5 min at 4 °C. The supernatants (cytoplasmic protein fractions) were kept at −80 °C. The pellets were resuspended in 100 µL of RIPA solution, vortexed, and incubated for 60 min on a rotator at 4 °C. Samples were centrifuged to insoluble pellet fraction at 13.000 rpm for 30 min at 4 °C and after the supernatants (nucleus protein fractions) were kept at −80 °C.

#### 2.1.7. Western Blot Analysis

Equal amounts of protein samples were subjected to SDS PAGE using 10% polyacrylamide gels under reducing conditions. Separated protein bands were transferred to nitrocellulose membranes in 10 mM CAPS (pH 11), containing 10% methanol. Membranes were blocked for 1 h at 4 °C with PBS containing 0.1% Tween 20 (PBS Tween) and 5% (*w*/*v*) low-fat milk powder. The membranes were incubated overnight at 4 °C on a rotating platform with the primary antibodies in PBS containing 0.1% Tween-20 (PBS-Tween) and 1% (*w*/*v*) low-fat milk powder. The immune complexes were detected following incubation with the appropriate peroxidase-conjugated secondary antibody diluted (1:5.000) in PBS-Tween, 2% low-fat milk for 1 h at room temperature, using the LumiSensor Chemiluminescent HRP substrate kit (Genscript; Piscataway, NJ, USA L00221V500), according to the manufacturer’s instructions. The protein expression of actin, tubulin (cytoplasmic marker), and lamin B1 (nuclear marker) was used to correct for the amount of each sample analyzed using ImageJ Analysis Software, National Institute of Health, Bethsesda, MD, USA.

#### 2.1.8. Immunofluorescence

MG63 cells were seeded on round coverslips placed in 24-well plates, at a concentration of 50.000 cells/well, and incubated in a complete medium for 24 h. Subsequently, the cells were incubated for 24 h at 37 °C and 5% CO_2_ in a 0% FBS medium. After treatment with biglycan (10 µg/mL) for 48 h, cells were fixed with 5% formaldehyde and 2% sucrose in PBS for 10 min at room temperature. Following 3 washes with PBS supplemented with 1% FBS, the permeabilizing agent Triton X-100 was added for 10 min and then washed before adding the primary antibody for 1 h at room temperature. Coverslips not incubated with the primary antibody were utilized as negative controls. The coverslips were rewashed and incubated for 1 h in the dark at room temperature with the appropriate cross-adsorbed secondary antibodies. TO-PRO-3iodide diluted 1:300 in de-ionized H2O was applied for 40 min for nuclear and chromosome counterstain. The coverslips were then placed onto slides using glycerol as a mounting medium and visualized using confocal microscopy. Pictures were analyzed using ImageJ Analysis Software.

#### 2.1.9. Statistical Analysis

Statistical significance was evaluated using a Student’s *t*-test, or one-way ANOVA analysis of variance with Tukey’s post-test, using GraphPad Prism (version 4.0) software.

### 2.2. Modeling Biglycan-IGF-IR Complexes

#### 2.2.1. Protein Structures

The model of the ectodomain of human Type 1 insulin-like growth factor receptor was obtained from the 3D crystallographic structure deposited in the PDB [PDB ID 5U8R, 3.00 Å] [40]. Five flexible loops were missing from the coordinates, and three of them could be modeled from homologous structures (38EDY and 155T-161M from PDB ID 1IGR, 2.60 Å [41], and 509D-516S from PDB ID 5U8Q, 3.27 Å [40,42], residue numbering correspond to 5U8R). The last two missing loops contained more than 20 residues (643Y-681A and 705P-723T) and were also absent in homologous structures. For these two loops, the SWISSMODEL server was applied [43], and a homology model based on 5U8R was used to obtain the structures of these regions.

Human biglycan was modeled from the 3D crystallographic structure of bovine biglycan [PDB ID 2FT3, 3.40 Å] [16], and 5 residues differing between bovine and human protein were mutated in AMBER LEaP (A47S, S254A, A258S, T273S, and V287M, residue numbering corresponds to 2FT3) [44]. Biglycans’ concave face is involved in the dimer formation for both biglycan and decorin. We, therefore, used a monomeric unit of biglycan in subsequent docking, allowing for the sampling of both faces in docking poses.

#### 2.2.2. Protein Structure Refinement of Homology Models

The final models of biglycan and receptor were subjected to 1 ns of molecular dynamics (MD) simulation, and the structures corresponding to the last frames from these trajectories were submitted to the docking server MD simulations were performed using AMBER 20 and the ff14SBonlysc force field [45,46]. Disulfide bridges were established for all relevant cysteine residues, and histidines were protonated on the ε-nitrogens (residue library denoted HIE in AMBER). Na+ counterions were added for the system net charge neutralization, and the structures were solvated in TIP3P octahedral periodic boxes with minimal distances of 4 Å to the periodic boundary. Energy minimization was performed in two steps, first with 0.5 × 103 steepest descent cycles and 103 conjugate gradient cycles, followed by 3 × 103 steepest descent cycles and 3 × 103 conjugate gradient cycles without restraints. Then, the system was heated to 300 K for 10 ps using a Langevin thermostat (γ = 1 ps^-1^), and equilibrated at 300 K and 105 Pa in the isothermic isobaric ensemble (NTP) for 100 ps, before a final production run for 20 ns in the same NTP ensemble. Solute atoms were subjected to harmonic force restraints of 100 kcal/mol/Å2 during the first minimization step and during heating. The SHAKE algorithm was used for all covalent bonds with hydrogens, and Particle Mesh Ewald method was used for treating electrostatics. The trajectories were visualized with VMD [47] and analyzed with the AMBER module CPPTRAJ.

#### 2.2.3. Electrostatic Potential Calculations

In order to assess if GAG chains are likely to be involved in the interaction, we calculated the electrostatic potential isosurface of the IGF-I receptor using the PBSA-program (Poisson–Boltzmann surface area) from AmberTools, AMBER16 [44], with a grid spacing of 1 Å. The surfaces were visualized with VMD, and the values for the positive and negative electrostatic potentials were optimized for visualization. Previously, it was shown that electrostatic potential calculations could be a powerful tool for GAG binding regions prediction [48].

#### 2.2.4. Docking Using the ClusPro Server

Biglycan and IGF-IR were docked using the ClusPro protein–protein docking server. Default parameters were used, and only models with balanced coefficient weights were considered [49].

#### 2.2.5. MD of Docked Complexes

The structural representatives of the five most populated clusters were prepared in LeaP (AMBER16) [44] from the ClusPro models. The starting structures were subjected to 20 ns of MD-simulation, utilizing a TIP3P octahedral periodic box boundary minimal distance to the solute of 8 Å and performed as described previously. After simulations, binding free energies and per residue decompositions of the complexes were estimated with the molecular mechanics-generalized Born surface area (MM-GBSA) method in AMBER16. Mode gb = 2 [50] for 200 evenly spaced frames for the 20 ns production run were used for calculations.

## 3. Results

### 3.1. Biglycan Forms a Complex with the Activated IGF-IR and Regulates Its Internalization and Nuclear Translocation

**Figure 1:**
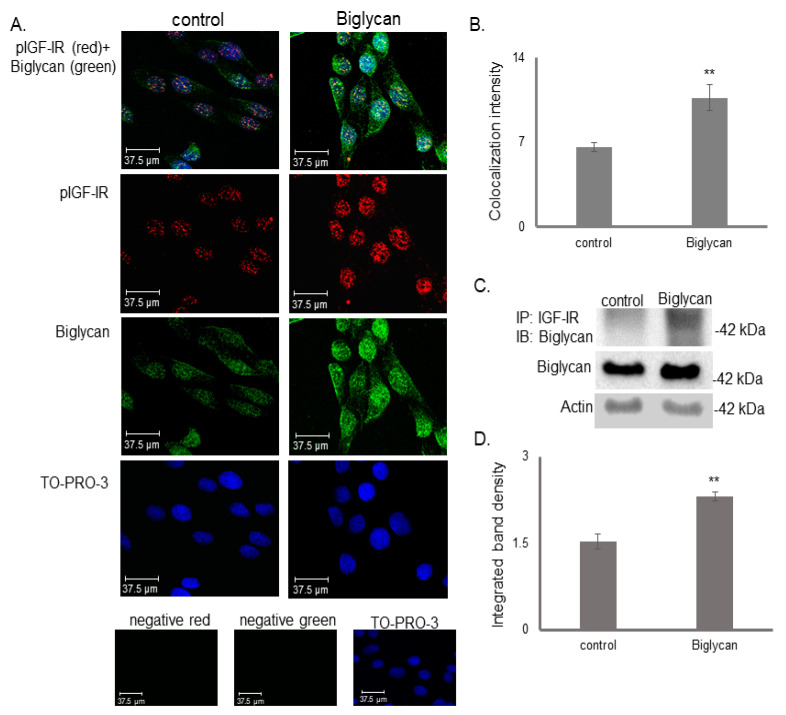
Co-localization of biglycan and pIGF-IR in MG63 cells. (**A**) Biglycan (green; anti-rabbit Alexa Fluor 488) and pIGF-IR (red; anti-mouse Alexa Fluor 555) protein staining of cells and respective nuclear staining (using TO-PRO-3) were evaluated in cultures after 48 h in serum-free medium (control) or biglycan (10 μg/mL). Primary antibodies were omitted in negative controls, but both secondary antibodies were used (anti-mouse—negative red; anti-rabbit—negative green). Slides were analyzed by confocal microscopy and pictures were taken using ×40 magnification. (**B**) Intensity measurement of co-localization of biglycan and pIGF-IR in MG63 cells was calculated using ImageJ Analysis Software. Representative pictures are presented. (**C**) Control cells were treated with serum-free culture medium, and samples were treated for 48 with biglycan (10 μg/mL). Cells extracts were incubated with IGF-IR antibody overnight in a rotating platform, and IGF-IR complexes were immunoprecipitated with Protein A/G. Western blot analysis was used to visualize biglycan protein immunoprecipitation with IGF-IR. (**D**) Densitometric analysis of the bands of immunoprecipitated proteins was normalized against the total expression of each protein in the cells and plotted. Representative pictures are presented. The results represent the average of three separate experiments. Means ± S.E.M. plotted; statistical significance is as follows: ** *p* ≤ 0.01, compared with the respective control samples.

We have previously shown that biglycan positively modulates poorly differentiated and biglycan-expressing MG63 osteosarcoma cell growth through an IGF-IR/β-catenin/ERK1/2 signaling conduit [30]. Considering the previously shown IGF-IR and biglycan co-operation in the regulation of MG63 cell growth, we examined their cellular localization. Utilization of immunofluorescence demonstrated deposition of pIGF-IR (red color) and biglycan (green color) in several different cell compartments, including membrane, cytoplasm, and surprisingly, nucleus. Furthermore, superimposition of the signals demonstrated a moderate colocalization of pIGF-IR and biglycan at these compartments (Figure 1A). Notably, treating the cells with recombinant biglycan increased the pIGF-IR and biglycan deposition and enhanced biglycan-pIGF-IR colocalization (Figure 1A,B).

We performed immunoprecipitation with an antibody specific for IGF-IR to verify immunofluorescence data. This approach confirmed the formation of a complex among these molecules, which was strongly enhanced after treating cells with exogenous biglycan (*p* ≤ 0.01) (Figure 1C,D). Since biglycan treatment does not affect IGF-IR total protein expression, the increased pIGF-IR/biglycan colocalization is attributed to enhanced IGF-IR activation due to biglycan action (Figure 1C,D).

**Figure 2:**
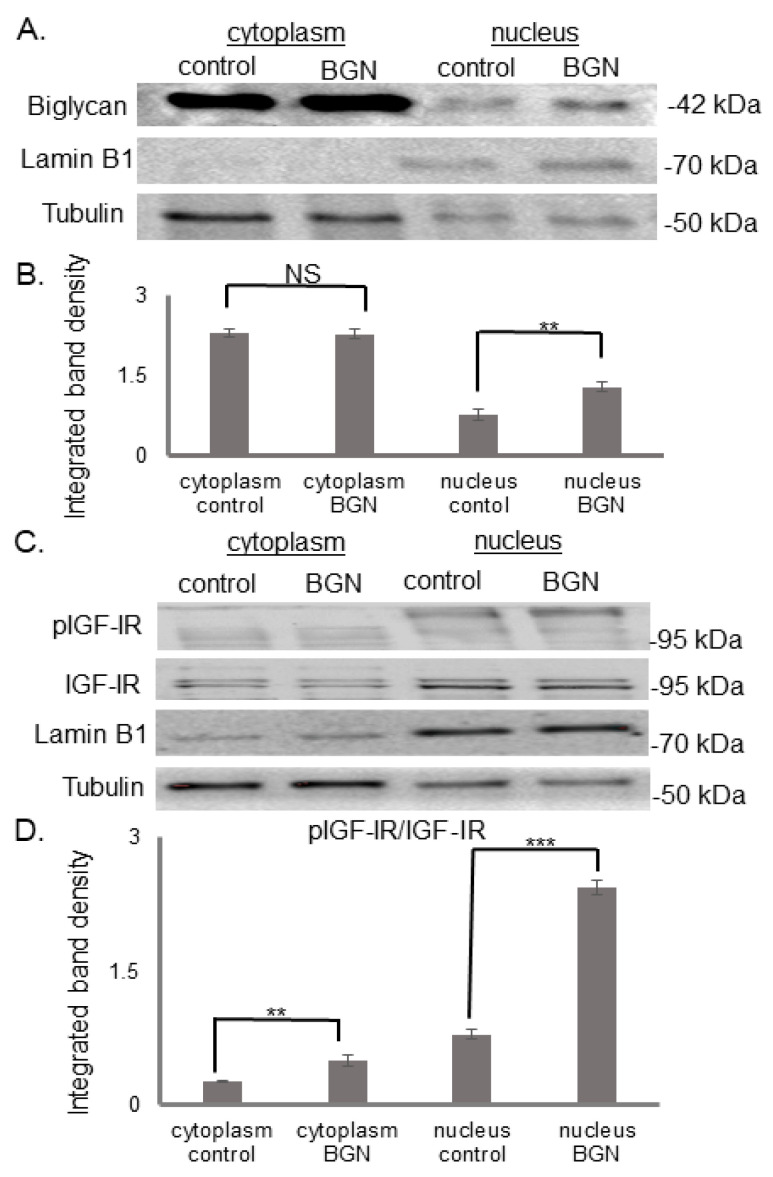
Effect of biglycan on its own deposition pIGF-IR protein expression at the different MG63 cell compartments. Expression of (**A**) biglycan and (**C**) pIGF-IR and in the cytoplasmic compartment of the cells treated with 0% FBS-medium (cytoplasm control) and cells treated with biglycan 10 μg/mL (cytoplasm BGN), as well as the nuclear compartment of the cells (nucleus control; nucleus BGN) were determined by Western blot analysis. Purity controls tubulin and lamin B1 were used for cytoplasmic and nuclear proteins, respectively. Equal amounts of protein from each compartment were loaded, and (**B**,**D**) densitometric analysis was performed and plotted. Representative blots are presented. Results represent the average of three separate experiments. Means ± S.E.M were plotted; statistical significance: ** *p* ≤ 0.01, *** *p* ≤ 0.001, not significant (NS) compared with the respective control samples.

Intriguingly, even though biglycan is predominantly a secreted molecule, immunofluorescence demonstrated that biglycan-treated cells exhibited enhanced biglycan deposition to the cell. Thus, extracts of biglycan treated MG-63 cells were collected, and cell fractions separated. Analyzing cell extracts with Western blot, as presented in Figure 2A,B, showed a significant increase in the deposition of biglycan to the nucleus (nucleus control vs. nucleus BGN; *p* ≤ 0.01), but not in the cytoplasm (*p* = NS), in biglycan treated in comparison to control cells. Furthermore, the analysis of the same protein extracts demonstrated that biglycan increases the internalization of activated IGF-IR to the cytoplasm (cytoplasm control vs. cytoplasm BGN; *p* ≤ 0.01) and its translocation to the nucleus (nucleus control vs. nucleus BGN; *p* ≤ 0.001) (Figure 2C,D). Therefore, these data suggest that biglycan regulates its localization and pIGF-IR deposition to different cell compartments.

#### 3.1.1. Modeling IGF-IR and Biglycan Structures

**Figure 3:**
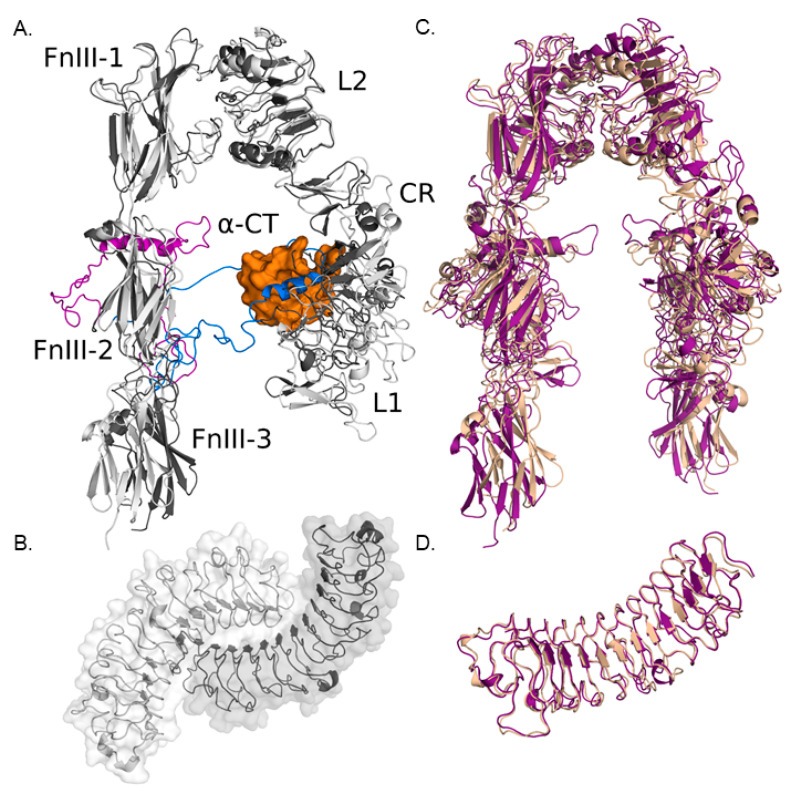
IGF-IR and biglycan models. (**A**) Aligned cartoon models of ectodomain monomers of apo-IGF-IR (in grey and purple) and IGF-IR complexed with IGF-I (in white and blue with IGF-I as orange surface). The colored loop regions are homology models from PDB IDs 5U8R and 5U8Q for apo-IGF-IR and IGF-IR complexed with IGF-I, respectively, created with SWISSMODEL [43]. (**B**) Bovine biglycan dimer (PDB ID 2FT3): the concave core region is unavailable for forming other interactions when biglycan is dimerized. (**C**,**D**) Aligned cartoon models of first (sand) and last (purple) frames from the 1 ns MD trajectory of the apo-IGF-IR and the human biglycan monomer models. The structures corresponding to the last frames were submitted to the ClusPro server for docking.

Protein structures of IGF-IR and biglycan were prepared to assess the putative sites of their docking. Two large loops in the crystal structure of the receptor lacked coordinates due to their high flexibility. The region between these two loops is a short α-helix, found in very different conformations in the crystal structures of the unbound receptor compared to the receptor bound to IGF (PDB IDs 5U8R and 5U8Q [1]) as shown in Figure 3A. This means that the flexibility of these loops is important for the conformational change upon IGF-binding. For these two loops, the SWISSMODEL server was applied [43], and a homology model based on 5U8R was used to obtain the structures of these regions (Figure 3A).

The resulting atomic fluctuations upon subjecting the receptor to 1 ns of MD simulation (Figure 3C and Figure S4) demonstrated that root means squared fluctuations (RMSF) of several regions moved up to 4 Å during the simulation. Despite this, the loop regions did not show significantly more flexibility than other parts of the receptor (residues 643Y-681A and 705P-723T, Figure S4). The part of the receptor that is modeled is only the ectodomain, and in vivo, the receptor would be anchored to the cell membrane, which could decrease the mobility of the ectodomain. Some observed fluctuations are probably, therefore, an artifact of modeling the free ectodomain only. The model used for the human biglycan monomer was more stable during the simulation than the receptor one (Figure 3D and Figure S3). Its concave face is involved in the dimer formation for both biglycan and decorin, and so when dimerized, it is unavailable for maintaining other interactions (Figure 3B).

#### 3.1.2. Computational Models of IGF-IR and Biglycan Docking

**Figure 4:**
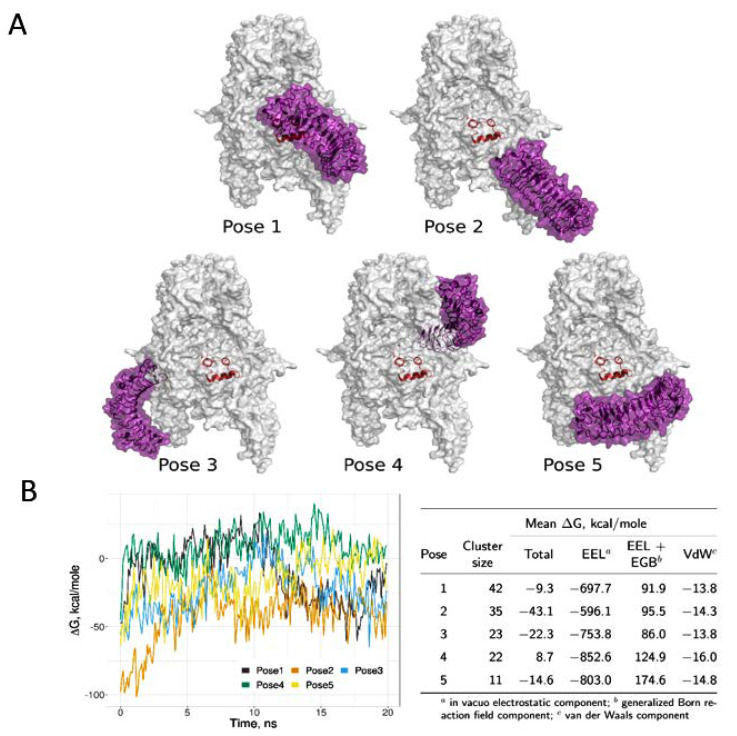
IGF-IR/biglycan possible binding sites. (**A**) The five binding poses of apo-IGF-IR (white) and human biglycan (purple) were obtained from ClusPro. IGF-I is shown in red cartoon to indicate its binding site within the receptor. The receptor structure used in docking was not complexed with IGF-I. (**B**) Total binding free energy (ΔG) obtained from MM-GBSA calculations from the 20 ns MD simulation is plotted for all five poses. The table lists the mean decomposed free energy components and the cluster sizes corresponding to each analyzed binding pose.

Docking with ClusPro yielded 30 poses, but after very similar poses based on visual inspection or poses bound symmetrically on the receptor monomers were excluded, we considered only 17 poses non-redundant. None of the suggested complexes had the convex face of biglycan interacting with the receptor, making it likely that biglycan is indeed interacting as a monomer through its concave side. In six non-redundant poses, only the C-terminus of biglycan interacted with IGF-IR, and of these six, two represented the most populated clusters. We did not, however, consider the C-terminal bound poses likely. A binding solely through its C-terminal would result in a very small binding interface, and this might result in unspecific binding. Moreover, in this case biglycan would not use its concave β-sheet face in the interaction. For decorin, this face is considered the most important in protein–protein interactions [51,52,53]. It cannot be ruled out that biglycan binds to the receptor through other interfaces, but we chose to exclude the C-terminal binding poses in further analysis due to their potentially unspecific nature. We analyzed the poses representing the five most populated ones of the remaining eleven clusters, shown in Figure 4A.

The five complexes were simulated for 20 ns, and MM-GBSA free energy calculations were performed. This allowed for a ranking of the total free energy of the complexes, and complex 2 had the most favorable binding free energy, followed by complex 3 (Figure 4B). Both of these complexes have biglycan bound to the lower part of the modeled receptor ectodomain, close to where the full-length receptor would be immersed in the cell membrane. Root mean squared deviation (RMSD), the measure of the structural stability of all five complexes during simulation, is shown in Figure S4.

None of the predicted biglycan poses suggest a competitive binding with IGF-I, as IGF-I is bound in between the receptor chains in a space too small for biglycan to access. In the most populated pose, pose 1 (with a cluster size from ClusPro of 42), biglycan is located close to the IGF-I binding site. Significant conformational changes are shown in this region between apo-receptor and IGF-I-bound receptor (PDB IDs 5U8R and 5U8Q, respectively) [40], and these changes might affect the interaction with biglycan. However, as our calculations do not point to this pose as the most energetically favorable, further analysis of this potential binding pose is beyond the scope of the present study.

From a biological aspect, to further investigate if IGF-I binding to its receptor affects biglycan binding, we performed a competition assay in which MG63 cells were treated with raising concentrations of IGF-I (10, 20, 40 ng/mL) in the absence or presence of biglycan. The results of this experiment indicated that IGF-I does not compete with biglycan, as this SLRP did not affect cells’ response to the growth factor (Figure S5).

#### 3.1.3. Effect of Biglycan Glycosylation on IGF-IR/Biglycan Docking

PBSA calculations showed that the negative electrostatic potential is predominant in the proximity of the receptor surface (Figure S6). These data make it unlikely that negatively charged GAG chains covalently linked to biglycan could be directly involved in the interaction. Notably, data on the interactions of structurally similar decorin show that the N-terminal GAG chains are not involved [52].

### 3.2. The Effect of Biglycan on Osteosarcoma Cell Growth Is Differentiation and Biglycan Expression Status Dependent

**Figure 5:**
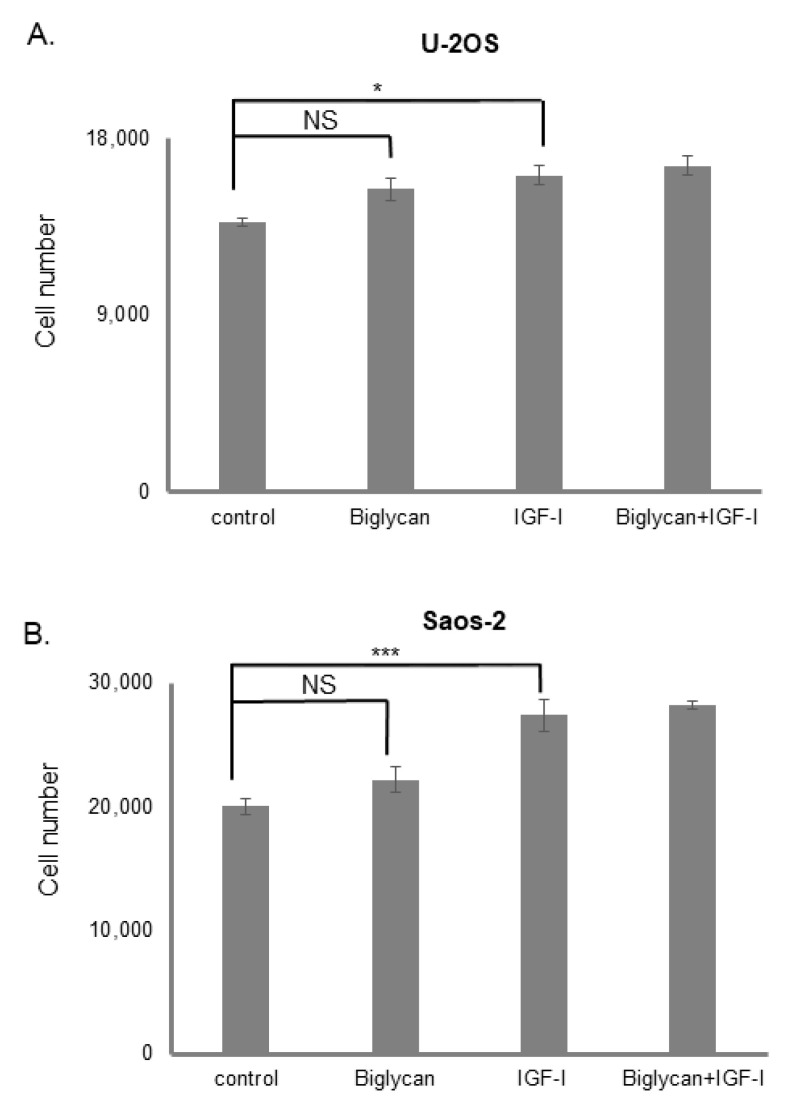
Effect of biglycan and IGF-I on (**A**) U-2OS and (**B**) Saos-2 cells proliferation. Cells were harvested and seeded (4000 cells/well for U-2OS and 20,000 cells/well for Saos-2) on 96-well plates and were allowed to rest overnight. The next day the medium was replaced with 0% FBS DMEM for 24 h. Biglycan treatment was added in 0% FBS medium for the next 48 h at 37 °C and 5% CO_2_. The cells were then lysed, and their number was calculated using the CyQUANT fluorometric assay kit. Results represent the average of three separate experiments. Means ± S.E.M were plotted; statistical significance: * *p* ≤ 0.05 *** *p* ≤ 0.001, not statistically significant (NS) compared with the respective control samples.

The MG63 cells are a poorly differentiated, aggressive osteosarcoma model expressing biglycan. Therefore, we aimed to assess whether osteosarcoma cells of different differentiation and biglycan expression status utilize the mentioned mechanism. For this reason, we used well-differentiated, biglycan non-expressing U-2OS [54] and Saos-2 [55] cells. As demonstrated in Figure 5, treatment with biglycan (10 μg/mL) did not affect basal or IGF-I-induced growth of U-2OS or Saos-2 cells. Therefore, in continuation, we focused on the mechanism of biglycans’ action in MG63 cells’ growth.

### 3.3. Biglycan Enhances IGF-IR Sumoylation

**Figure 6:**
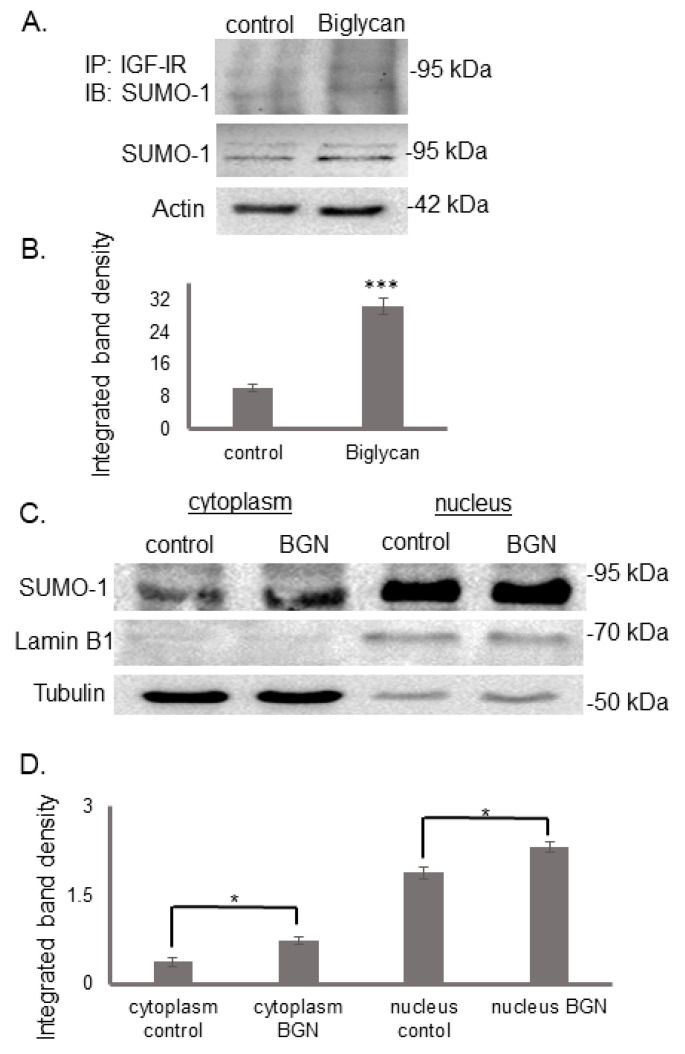
Effect of biglycan on colocalization of IGF-IR with SUMO-1 and SUMO-1 deposition in the cell. Control cells were treated with serum-free culture medium, and samples were treated for 48 with biglycan (10 μg/mL). (**A**) Cells extracts were incubated with IGF-IR antibody overnight in a rotating platform and IGF-IR complexes were immunoprecipitated with Protein A/G. Western blot analysis was used for the visualization of SUMO-1 protein immunoprecipitation with IGF-IR. (**B**) Densitometric analysis of the bands of immunoprecipitated proteins was normalized against the total expression of each protein in the cells and plotted. (**C**) Expression of SUMO-1 in the cytoplasmic compartment of the cells treated with 0% FBS-medium (cytoplasm control) and cells treated with biglycan 10 μg/mL (cytoplasm BGN), as well as the nuclear compartment of the cells (nucleus control; nucleus BGN) were determined by Western blot analysis. Purity controls tubulin and lamin B1 were used for cytoplasmic and nuclear proteins, respectively. Equal amounts of protein from each compartment were loaded, and (**D**) densitometric analysis was performed and plotted. Representative blots are presented. Results represent the average of three separate experiments. Means ± S.E.M were plotted; statistical significance: * *p* ≤ 0.05, *** *p* ≤ 0.001 compared with the respective control samples.

It is well established that the IGF-IR is predominantly localized at the cell membrane, although significant cytoplasmic and nuclear levels might be observed [56]. However, as IGF-IR lacks a nuclear localization signal, it needs an additional structural motif such as the conjugation with the small ubiquitin-like modifier-1 (SUMO-1) [57]. Cell fractionation and Western blot analysis revealed the presence of SUMO-1 in the cytoplasm and the nucleus of MG63 cells. Furthermore, the localization of SUMO-1 in these cell compartments was strongly enhanced after biglycan treatment (cytoplasm control vs. cytoplasm BGN; *p* ≤ 0.05, nucleus control vs. nucleus BGN; *p* ≤ 0.05) (Figure 6C,D). In continuation, we wanted to assess putative IGF-IR/SUMO-1 colocalization. Immunoprecipitation and Western blot detected an IGF-IR/SUMO-1 complex whose expression was enhanced in biglycan-treated cells (*p* ≤ 0.001) (Figure 6A,B). These data suggest that biglycan enhances IGF-IR sumoylation and respective nuclear translocation.

### 3.4. IGF-IR Colocalizes with DNA in a Biglycan-Dependent Manner

**Figure 7:**
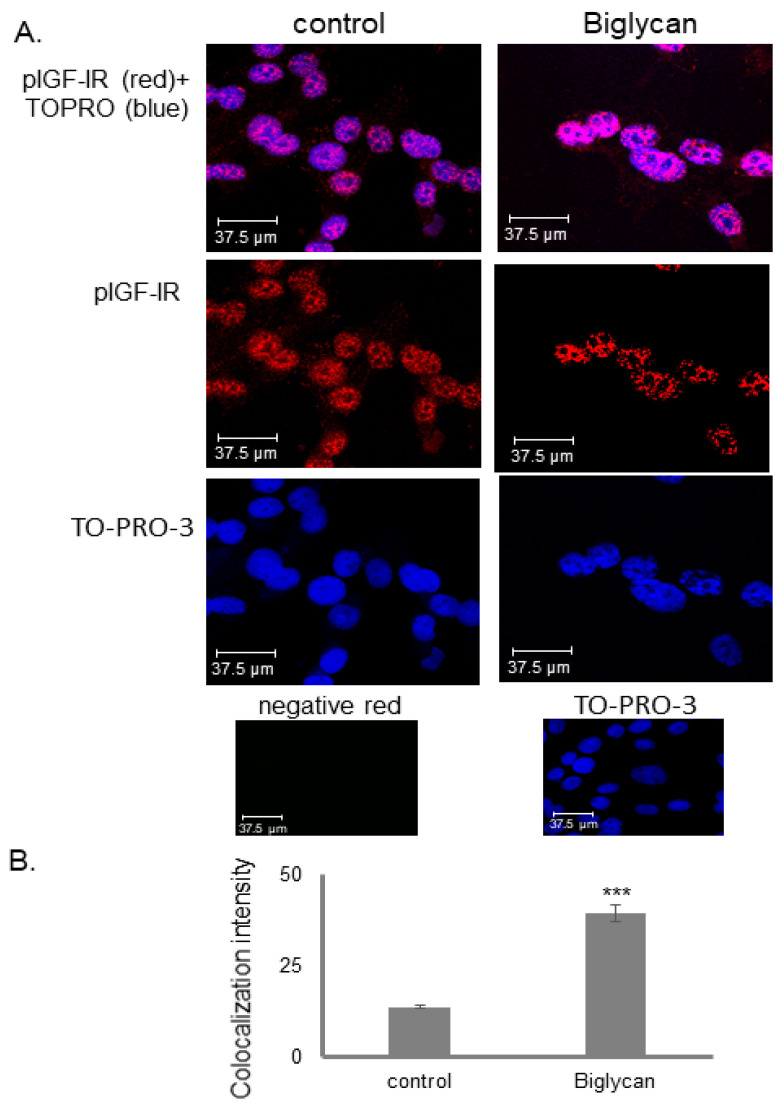
Co-localization of pIGF-IR with DNA in MG63 cells. (**A**) pIGF-IR(red; anti-mouse Alexa Fluor 488) protein staining of cells and respective nuclear staining (using TO-PRO-3) were evaluated in cultures after 48 h in serum-free medium (control) or biglycan (10 μg/mL). In negative controls, primary antibody was omitted, but secondary antibody was used (anti-mouse negative red). Slides were analyzed by confocal microscopy and pictures were taken using ×40 magnification. (**B**) Intensity measurement of colocalization of pIGF-IR with DNA was calculated using ImageJ Analysis Software. Representative pictures are presented. Results represent the average of three separate experiments. Statistical significance: *** *p* ≤ 0.001 compared with the respective control samples.

Nuclear IGF-IR has been characterized as a co-transcriptional factor which, in synergy with the LEF1 transcriptional regulator, increases the expression of LEF1 downstream target genes, such as cyclin D1 [58]. Our previous results had demonstrated that biglycan enhances cyclin D1 expression [30]. So, to assess if nuclear IGF-IR acts putatively as a transcriptional regulator in our OS model, we examined its DNA binding with immunofluoresence. In the nucleus, the interaction of pIGF-IR (red color) with DNA, stained with TOPRO-3 (blue color), was studied. Colocalization of the activated nuclear IGF-IR receptor with DNA regions was detected (purple stain). Notably, this interaction was more enhanced in biglycan-treated cells suggesting that biglycan promotes IGF-IR transcriptional activity (Figure 7).

### 3.5. The Tyrosine Kinase Activity of IGF-IR Affects Its’ Cellular Localization and Sumoylation

**Figure 8:**
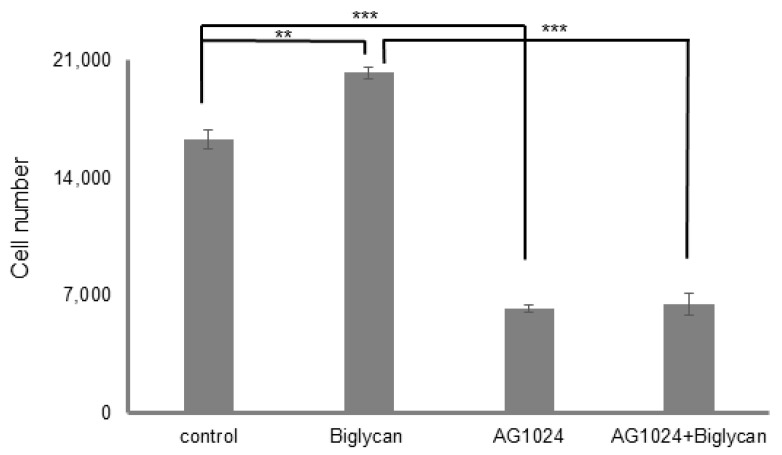
Role of IGF-IR on biglycan-dependent MG63 cell proliferation. MG63 cells were harvested and seeded on 96-well plates and were allowed to rest overnight. The next day, cells were treated with serum-free medium for 24 h. Cells, in each well, incubated with 0% FBS-medium (control), 10 μg/mL biglycan, 10 µM AG1024 and 10 μg/mL biglycan + 10 µM AG1024 for 48 h (30 min pre-treatment). Cells were counted using fluorometric CyQUANT assay kit. Results represent the average of three separate experiments. Means ± S.E.M were plotted; statistical significance: *** *p* ≤ 0.001, ** *p* ≤ 0.01 compared with the respective control samples.

MG63 cells were treated with AG1024 (10 μM), a substrate competitive, specific IGF-IR inhibitor for 48 h, with the presence or absence of biglycan (10 μg/mL) to further assess the role of IGF-IR on the biological actions of biglycan. Biglycan-dependent proliferation of MG63 cells is completely abolished in AG1024-treated cells, confirming the vital role of the receptor in biglycan-related growth mechanism (Figure 8).

**Figure 9:**
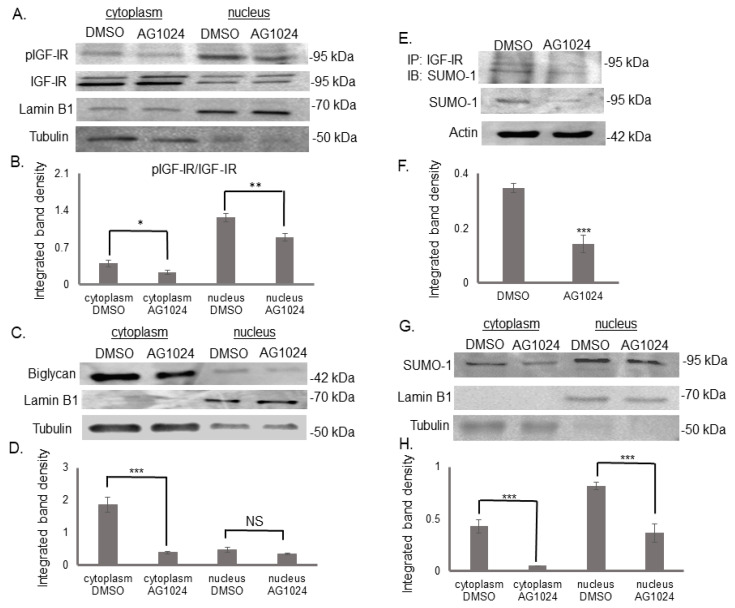
IGF-IR activation as an obligatory step of the mechanism. Expression of (**A**) pIGF-IR, (**C**) biglycan, and (**G**) SUMO-1 in the cytoplasmic compartment of the cells treated with DMSO in 0% FBS-medium (cytoplasm DMSO) and cells treated with AG1024 10 μM (cytoplasm AG1024), as well as the nuclear compartment of the cells (nucleus DMSO; nucleus AG1024) were determined by Western blot analysis. Purity controls tubulin and lamin B1 were used for cytoplasmic and nuclear proteins, respectively. (**E**) Cells extracts were incubated with IGF-IR antibody overnight in a rotating platform and IGF-IR complexes were immunoprecipitated with Protein A/G. Western blot analysis was used for the visualization of SUMO-1 protein immunoprecipitation with IGF-IR. Equal amounts of protein were loaded and (**B**,**D**,**F**,**H**) densitometric analysis was performed and plotted. Representative blots are presented. Results represent the average of three separate experiments. Means ± S.E.M were plotted; statistical significance: * *p* ≤ 0.05, ** *p* ≤ 0.01, *** *p* ≤ 0.001, not statistically significant (NS) compared with the respective control samples.

MG63 cells were also treated with AG1024 to assess whether IGF-IR cell localization depends on its tyrosine kinase activity. The assessment of IGF-IR localization by immunoprecipitation and Western blot detected a decrease in the levels of activated receptor, both in the cytoplasm and the nucleus in treated compared with the control cells (cytoplasm DMSO vs. cytoplasmAG1024; *p* ≤ 0.05, nucleus DMSO vs. nucleus AG1024; *p* ≤ 0.01) (Figure 9A,B)

In addition, as presented in Figure 9E,F, IGF-IR tyrosine kinase activity inhibition significantly decreased its sumoylation (DMSO vs. AG1024; *p* ≤ 0.001). Furthermore, AG1024 treatment reduced the translocation of the sumoylated IGF-IR complex both to the cytoplasm (cytoplasm DMSO vs. cytoplasm AG1024; *p* ≤ 0.001) and to the nucleus, as demonstrated by Western blot and immunoprecipitation (nucleus DMSO vs. nucleus AG1024; *p* ≤ 0.001) (Figure 9G,H). Collectively, these results suggest that the biglycan-dependent IGF-IR internalization and nuclear translocation are dependent on IGF-IR activation.

### 3.6. Biglycan Promotes MG63 Osteosarcoma Cells’ Aggressive Phenotype

**Figure 10:**
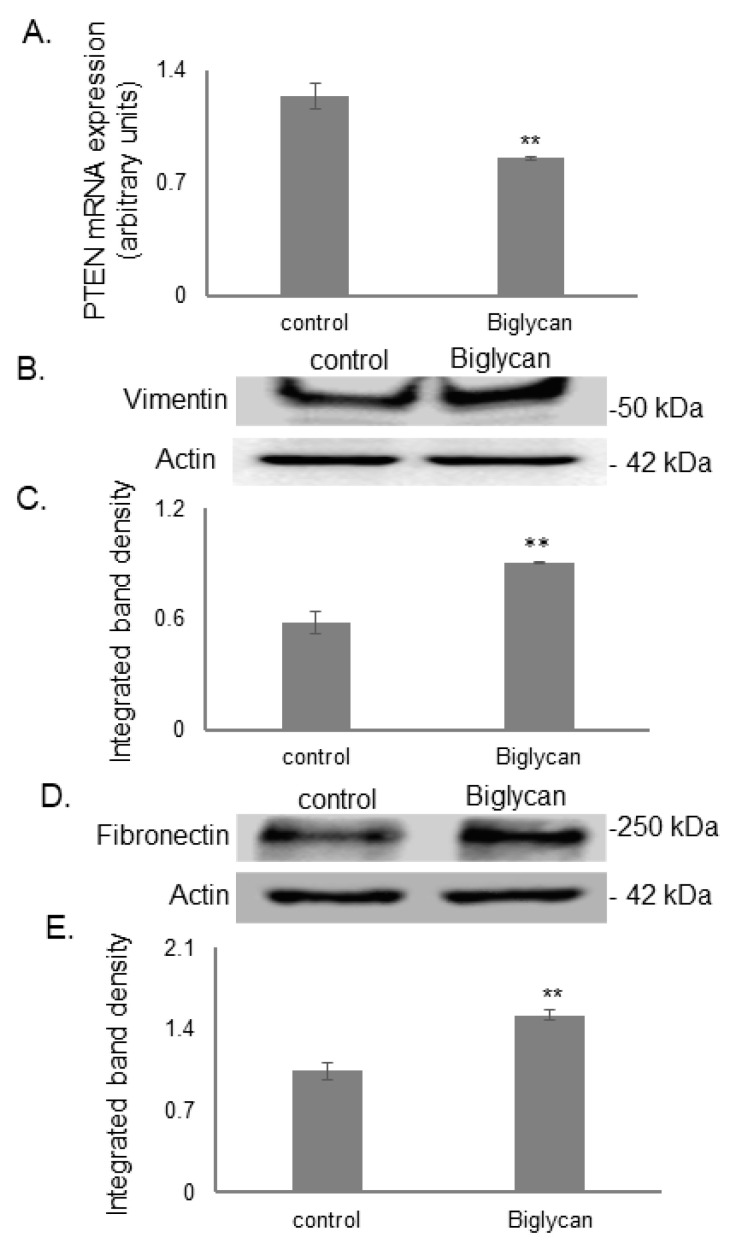
Effect of biglycan on PTEN expression and aggressiveness markers, vimentin, and fibronectin expression. (**A**) PTEN mRNA levels in MG63 cells treated with biglycan (10 μg/mL) during 48 h were determined by real time PCR using primers specific for the PTEN gene and normalized against GAPDH. (**B**) Vimentin and (**D**) fibronectin in cells treated with serum-free medium (control) and cells treated with biglycan (10 μg/mL) was determined by Western blot analysis. (**C**,**E**) Densitometric analysis of the protein bands of vimentin and fibronectin were normalized against actin and plotted. Representative blots are presented. Results represent the average of three separate experiments. Means ± S.E.M were plotted; statistical significance: ** *p* ≤ 0.01, compared with the respective control samples.

Biglycan has been shown to induce IGF-IR/ERK 1/2 signaling in osteosarcoma cells [30]. Deregulation of these vital signaling pathways can result in tumor progression associated with the loss or decrease in a tumor-suppressor gene, PTEN, and enhanced expression of tumor aggressivity markers [59,60]. Notably, biglycan treatment in our osteosarcoma model attenuated PTEN mRNA expression levels (Figure 10A; *p* ≤ 0.05). Furthermore, Western blot showed that the protein expression of aggressiveness markers, vimentin, and fibronectin was increased in biglycan-treated cells (Figure 10B–E; *p* ≤ 0.01).

### 3.7. Biglycan Controls Chemosensitivity to Doxorubicin in MG63 Osteosarcoma Cells

**Figure 11:**
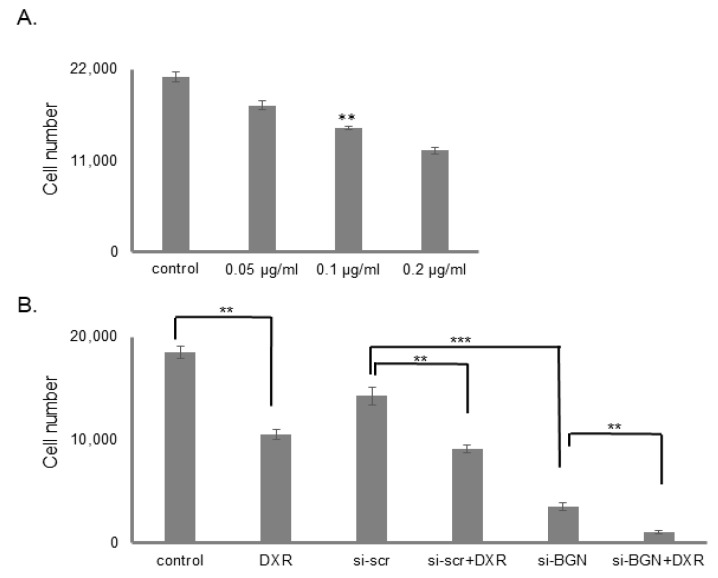
Effect of biglycan and doxorubicin on MG63 cells proliferation. MG63 cells were harvested and seeded (1500 cells/well) on 96-well plates and transfection with siRNAs (short interfering RNAs) was performed, when needed. (**A**) Control cells were treated with completed culture medium and samples were treated with different doxorubicin concentrations for 48 h. (**B**) Cells, in each well, were incubated in serum-free medium and transfected with either siRNAs against biglycan (siBGN) or scrambled siRNAs (siScr), used as negative control. After 24 h, the medium was replaced with 10% FBS DMEM and cells were incubated with 0.1 μg/mL doxorubicin for 48 h. Cells, in each well, were counted after an incubation period, using fluorometric CyQUANT assay kit. Results represent the average of three separate experiments. Means ± S.E.M were plotted; statistical significance: ** *p* ≤ 0.01, *** *p* ≤ 0.001 compared with the respective control samples.

Doxorubicin is used for osteosarcoma treatment [61]. However, its administration in osteosarcoma cells is correlated to mechanisms of chemotherapy resistance [62]. In addition, biglycan has previously been shown to negatively affect tumor cell therapy response in OS and other tumors [39,63]. Therefore, we examined the putative effects of biglycan on MG63 cells’ response to doxorubicin. Treatment of the osteosarcoma cells during 48 h with different concentrations of doxorubicin induced a concentration-dependent decrease in the MG63 cell number as established using the CyQUANT fluorometric assay (Figure 11A; *p* ≤ 0.001). Biglycan-deficient MG63 cells (si-BGN) were generated to estimate if biglycan may affect their response to doxorubicin. Treatment of siBGN and control scrambled si RNA cells (si-scr) with doxorubicin (0.1 μg/mL) for 48 h showed that the attenuation of cell growth by doxorubicin was enhanced in biglycan-deficient cells (Figure 11B; *p* ≤ 0.01). Therefore, biglycan exhibits doxorubicin protective effects in MG63 osteosarcoma cells.

## 4. Discussion

Biglycan regulates critical cellular functions, including matrix assembly, cellular migration and adhesion, cell growth, and apoptosis; thus, not surprisingly, alterations in its expression are correlated to carcinogenesis [24,64]. Indeed, biglycan is shown to upregulate VEGF expression in colon cancer cells and promote tumor angiogenesis [65]. Moreover, biglycan has been characterized as a danger-associated molecular pattern (DAMP). It has been suggested to regulate the crosstalk between inflammation and autophagy by evoking a switch between pro-inflammatory CD14 and pro-autophagic CD44 co-receptors for TLRs relevant to cancer progression [64]. Furthermore, biglycan has been suggested to regulate the function of antigen-presenting cells, including macrophages and dendritic cells, with implications in cancer [66].

Biglycan is an important component of the bone osteoid [67]. The role of biglycan in osteosarcoma is not well established even though the expression of this SLRP changes in osteosarcoma, partly correlated to the cancer differentiation status. Some reports implicate biglycan as the crosstalk between PTH(1–34), and FGF-2 signaling alters biglycan ECM content regulating osteosarcoma cell migration [68]. In addition, biglycan was shown to control osteosarcoma cell proliferation through a LRP6/β-catenin/IGF-IR signaling axis [30]. Interestingly, the cAMP/protein kinase A signal transduction pathway was found to enhance biglycan expression in osteosarcoma cells [69].

The IGF-I/IGF-IR signaling pathway is well-established to exert a pro-oncogenic effect in sarcomas [11,13]. Indeed, Wang et al., by implementing tissue microarray analysis, associated poor prognosis with high expression of IGF-I in osteosarcoma patients [70]. Furthermore, a meta-analysis demonstrated a generalized IGF-IR signaling input in sarcoma progression, relating IGF-IR expression with poor outcomes in sarcoma patients [32].

Previously, we reported that IGF-I increases biglycan expression in MG63 osteosarcoma cells [30]. Notably, the present study shows that biglycan activates the IGF-IR signaling pathway by forming a complex with the receptor. Biglycan has a 55% sequence identity with decorin and a similar structure [16,71]. Decorin has been shown to bind to a variety of biological mediators, including growth factor receptors, and it is suggested that it is the concave face of decorin, containing 14 curved β-sheets, that is involved in most of its interactions [51,52,53]. Well in accordance with these data, our docking models suggest that biglycan binds the receptor via its concave surface.

The previously shown synergistic effect between biglycan and IGF-I on cell growth suggests the two ligands should not compete for binding. Indeed, none of the predicted docking poses suggest a competitive binding, as IGF-I is bound in between the receptor chains in a space too small for biglycan to access. In the two most stable complexes in terms of binding energy, biglycan is bound to the lower part of the modeled receptor ectodomain, close to where the full-length receptor would be immersed in the cell membrane. Moreover, its glycan chains do not appear to participate in this interaction. In a previous report, biglycan was shown to bind with a high affinity to CD14 via its protein core, which agrees with the results of the present study [72].

Indeed, biglycan binding was shown in the current study to result in prolonged IGF-IR activation leading to protracted IGF-IR-dependent OS cell growth response. Interestingly, biglycan facilitates the internalization and nuclear translocation of IGF-IR in our OS model. Notably, IGF-IR non-canonical signaling is emerging as an important signaling axis. Indeed, three potential outcomes have been suggested for internalized IGF-IR: (i) localization to the plasma membrane; (ii) degradation or (iii) nuclear translocation [73]. The first evidence of IGF-IR nuclear localization was shown for renal epithelial cells [74]. Importantly, significant IGF-IR nuclear localization has been shown in several cancer types, including renal and breast cancers and advanced or malignant prostate cancer cells [75]. Moreover, these authors determined that nuclear IGF-IR was robustly associated with the advanced tumor stages of prostate cancer patients.

Previously, it has been shown that nuclear translocation of IGF-IR is dependent on its activation status and is facilitated by IGF-I and -II [76]. Moreover, the intact activated receptor is suggested to engage in nuclear trafficking [76]. Similarly, in the present study, the translocation of IGF-IR was strongly inhibited in the presence of a specific IGF-IR inhibitor, showing that receptor activation is obligatory for its subsequent nuclear localization.

Previously, sumoylation was shown to exert a key role in the nuclear translocation of the IGF-IR [57]. Moreover, the sumoylation was dependent on receptor phosphorylation status, as shown through the utilization of specific inhibitors. In the present study, the internalized receptor was demonstrated to undergo sumoylation in a biglycan-dependent manner. Since biglycan enhances and protracts receptor activation, these data suggest that it also facilitates subsequent receptor sumoylation. Notably, biglycan enhanced both cytoplasmic and nuclear SUMO-1 content.

Earlier studies suggest that IGF-IR can be uptaken by caveolin- and clathrin-mediated endocytosis, which includes a recycling pathway [57,77]. Furthermore, various mechanisms for IGF-IR nuclear trafficking have been implicated, considering that IGF-IR lacks a nuclear localization sequence (NLS) sequence. Thus, Warsito et al., suggest that RanBP2, a SUMO E3 ligase located at the nuclear pore complex, can bind IGF-IR. Likewise, importin-β, correlated to nuclear trafficking, was also shown to colocalize with IGF-IR, as does α-tubulin [58]. However, the mechanisms responsible for its nuclear translocation remain undefined [78]. Notably, biglycan contains potential nuclear localization signals, suggesting that it is involved in regulating cellular functions by directly participating in nuclear processes [79,80]. Therefore, biglycan-IGF-IR complex translocation to the nucleus could be elicited by biglycans’ NLS sequence [79,81].

IGF-IR was shown to interact with double-stranded DNA [57] physically. Moreover, IGF-IR binding to chromatin was shown to depend on its activation status [82]. In the present study, biglycan enhances nuclear IGF-IR binding to genomic DNA and possibly promotes receptor’s transcriptional activity. Warsito et al. have shown that the nuclear IGF-IR receptor is associated with TCF/LEF transcriptional factors in the cell nucleus and facilitates the transcription of target genes, such as the cell-cycle regulator Cyclin D1 [58]. Interestingly, biglycan-deficient MG63 cells express lower levels of activated IGF-IR and Cyclin D1 [30], supporting the hypothesis that biglycan enhances IGF-IR activation, intracelularization, nuclear translocation, and transcriptional activity axis.

The phosphatase and tensin homolog gene (PTEN) is one of the most frequently altered genes in osteosarcoma [83]. PTEN is a crucial tumor suppressor gene inhibiting phosphatidylinositol 3-kinase (PI3K)/protein kinase B (AKT)/mammalian target of rapamycin (mTOR) pathway, which is often aberrant in cancer [59]. Notably, the loss or decrease in PTEN expression has been demonstrated to be associated with a high risk of metastasis and dismal outcomes in osteosarcoma patients [59]. Previously, IGF-IR and PTEN expression were shown to be oppositely regulated in a manner correlated to carcinogenesis [84], which was confirmed in a clinical study with prostate cancer patients [85]. Our results showed that biglycan attenuates PTEN expression. Downregulation of PTEN in vitro and in vivo upregulates EMT markers and promotes osteosarcoma metastasis [59,86]. Moreover, the present study confirms an increased expression of aggressiveness markers vimentin and fibronectin in MG63 biglycan treated cells.

The oncogenic role of IGF-I/IGF-IR signaling in sarcomas is well established [11]. Furthermore, upregulation of IGF-I/IGF-IR signaling is associated with the mechanisms of chemoresistance as the pIGF-IR expression is increased in various drug-resistant cancers [87,88,89]. Indeed, the different mechanisms of this activation contribute to cancer cells proliferation and induce resistance by over-activating Grb2/RAS/RAF/MAPK cascades [90]. In gastric cancer patients, overexpression of IGF-IR is associated with poorer chemotherapy outcomes in comparison with patients with low expression of the receptor [91]. In a colorectal cancer model, it is shown that IGF-IR nuclear translocation is associated with resistance to chemotherapy and targeted therapies both in vitro and clinically in patients [92].

Notably, biglycan is one of nine outstandingly differentially expressed osteosarcoma genes between responders and poor responders to chemotherapy [39]. Furthermore, biglycan was shown to upregulate MG63 osteosarcoma cells ERK 1/2 activation [30], previously correlated to their chemoresistance profile [39]. Moreover, separate bioinformatics analysis revealed that ECM organization genes are differentially expressed between doxorubicin-resistant human osteosarcoma cell line MG63/DXR and its parental cell line MG63 [93]. Our present data show that MG63 cells’ response to the osteosarcoma anticancer agent doxorubicin [94] is enhanced by biglycan. Thus, biglycan protects the aggressive MG63 cells against doxorubicin effects.

To better understand this mechanism, we tested if biglycan affects the proliferation of the biglycan non-expressing U-2OS and Saos-2 osteosarcoma cells. Indeed, Benayahu et al., have shown that mRNA for biglycan was detected only in primary cells and MG63 cell line and was undetectable in RNA from U-2OS and Saos-2 osteosarcoma cell lines [3]. In our study, U-2OS and Saos-2 cells did not respond to biglycan action (Figure 5). Another difference between these osteosarcoma cell lines is the mutation status of the oncosuppressive gene p53, which is required for cell cycle arrest or apoptosis. U-2OS is the wild-type cell line, Saos-2 is a mutant cell line, and MG63 is a null cell line regarding p53 expression/activity [95,96]. Further studies in other osteosarcoma models, in vivo models, and patient biopsies could provide more refined data on the mechanism presented here.

## 5. Conclusions

**Figure 12:**
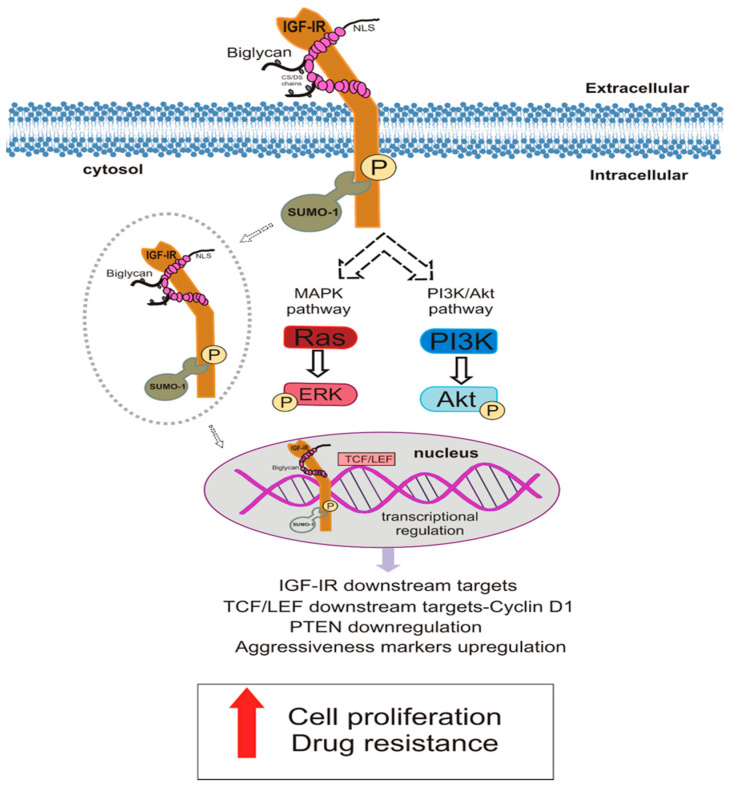
Biglycan interacts with IGF-IR to regulate osteosarcoma cells behavior. Biglycan binds to IGF-IR and activates the signaling cascade of the receptor. This complex formation stimulates the IGF-IR uptake to the cytoplasm, its sumoylation, and deposition to the nucleus where the receptor, putatively, acts as a transcriptional regulator or target genes. This mechanism involves biglycan in prolonged IGF-IR activation. The synergistic action of these molecules increases MG63 osteosarcoma cells aggressiveness, leading to enhanced growth and chemoresistance.

In summary, we report for the first time that biglycan, a class I SLRP, can bind to IGF-IR and enhance the growth and aggressivity markers of MG63 osteosarcoma cells. The schematic presentation of the proposed mechanism is shown in Figure 12. Whereas failures have been registered in creating novel targeted therapeutics regarding sarcomas, new agent development, evaluating combinatorial strategies for enhancing antitumor responses, and better classifying the patients with more specific biomarkers could improve the therapeutic approach and patient outcome. An approach for developing a combinatorial strategy is to focus on the tumor microenvironment, which transfers various signals into cancer cells and changes their behavior. Herewith, we propose a potential direct and adjunct therapeutical implication of the ECM effector biglycan in osteosarcoma.

## Data Availability

The data presented in this study are available in this article (and supplementary material).

## Supplementary Materials

The following supporting information can be downloaded at: https://www.mdpi.com/article/10.3390/cancers14051196/s1, Figure S1. Optimization of cell number seeding for proliferation assay. Figure S2. RMSD of five possible complexes of IGF-IR/biglygan. Figure S3. Electrostatic potential in the proximity of the receptor surface. Figure S4. RMSD of five possible complexes of IGF-IR/biglycan. Figure S5. Competition assay between biglycan and IGF-I on MG63 cell proliferation effect. Figure S6. Electrostatic potential in the proximity of the receptor surface. Supplementary materials and methods.
