# Supplementary material for: Biglycan Interacts with Type I Insulin-like Receptor (IGF-IR) Signaling Pathway to Regulate Osteosarcoma Cell Growth and Response to Chemotherapy
Source: Cancers (Basel). 2022 Feb 25;14(5):1196. doi: 10.3390/cancers14051196 (PMC8909324; doi:10.3390/cancers14051196)
Supplement: Supplementary file 1 [file cancers-14-01196-s001.zip › cancers-1562188-SUPPLEMENTARY MATERIAL .pdf]

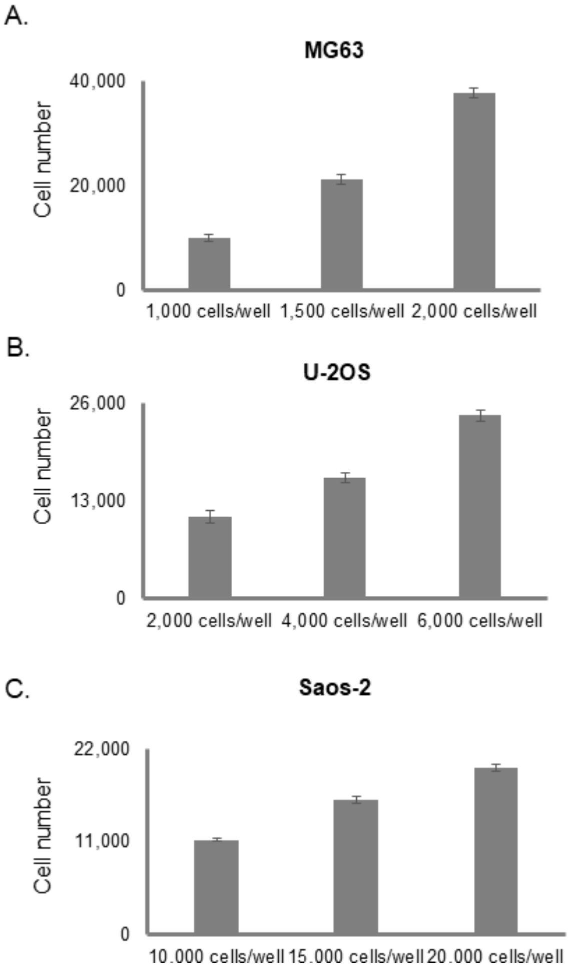

Figure S1. Optimization of cell number seeding for proliferation assay. Cells were harvested and seeded on 96-well plates at different cell concentrations and were allowed to rest overnight. (A) MG63 cells (B) U-2OS and (C) Saos-2 were incubated in a serum-free medium for 72h. Cells in each well were counted after the incubation period, using a fluorometric CyQUANT assay kit. Results represent the average of three separate experiments.

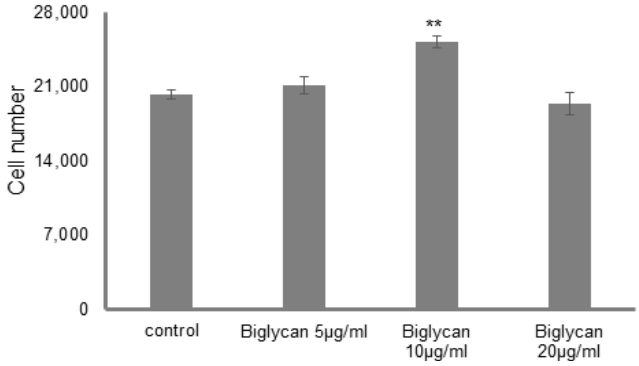

Figure S2. Optimization of biglycan concentration. Cells were harvested and seeded on 96-well plates and were allowed to rest overnight. The next day, cells were treated with a serum-free medium for 24h. MG63 cells were treated with raising concentrations of biglycan (5, 10, 20 μg/mL) in a serum-free culture medium for 48h. Cells in each well were counted after the incubation period, using a fluorometric CyQUANT assay kit. Results represent the average of three separate experiments. Means ± S.E.M were plotted; statistical significance: **$p \leq 0.01$ compared with the respective control samples.

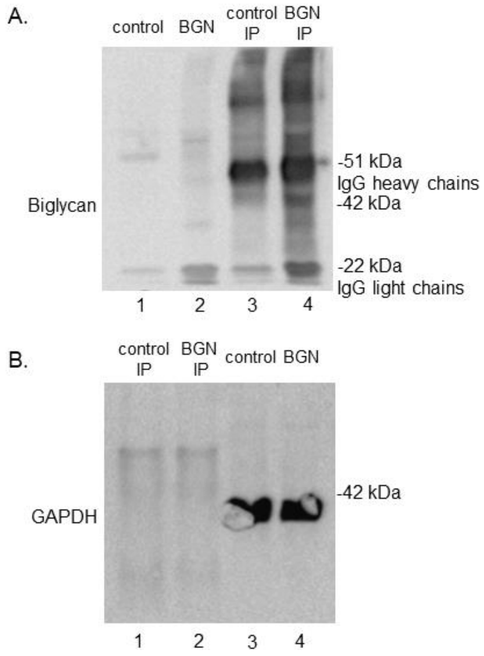

Figure S3. Immunoprecipitation controls. Cells treated in serum-free medium (control) and cells treated with biglycan (10 μg/mL) (BGN) were lysed. (A) Cell extracts, loaded in lanes 1 and 2, were not incubated with IGF-IR antibody, while cells extracts, loaded in lanes 3 and 4, were incubated with IGF-IR antibody overnight. The next day, protein A/G with agarose was added, and the samples were utilized for Western blot analysis. (B) In lanes 1 and 2, the samples contained immunoprecipitated proteins while, in lanes 3 and 4, total cell extracts from treated cells were used for Western blot analysis.

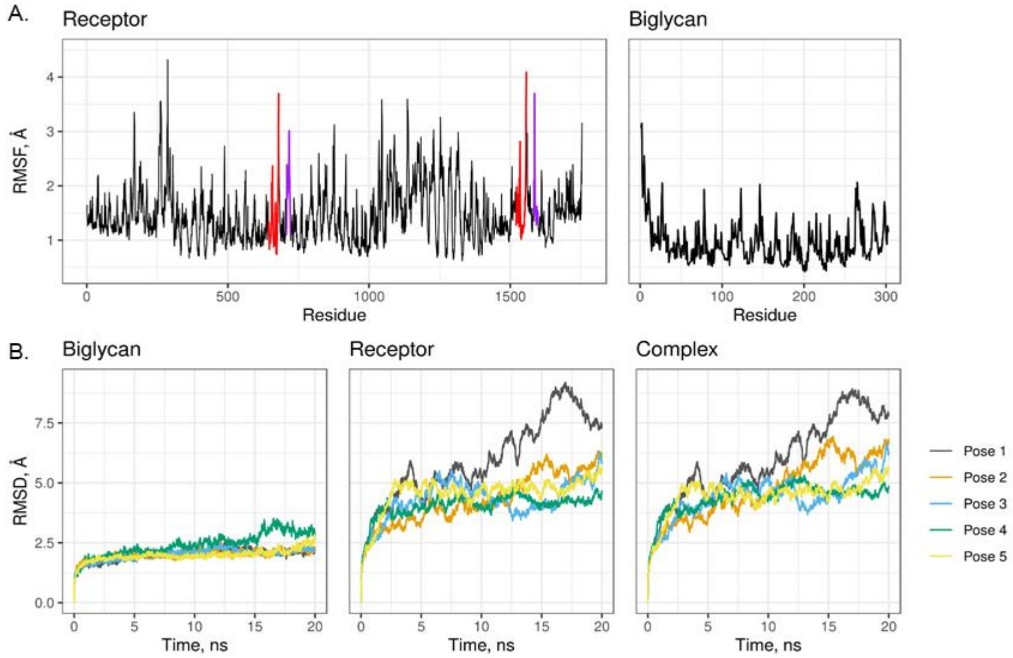

Figure S4. RMSD of five possible complexes of IGF-IR/biglycan. (A) Root mean square fluctuations (RMSF) of residues in the models of IGF-1R and biglycan during one ns MD simulations. The two modeled loop regions are shown in red (residues 643-681) and purple (residues 705-723) for both receptor monomers. (B) Root mean square deviations (RMSD) for the five docked poses during 20 ns MD simulation.

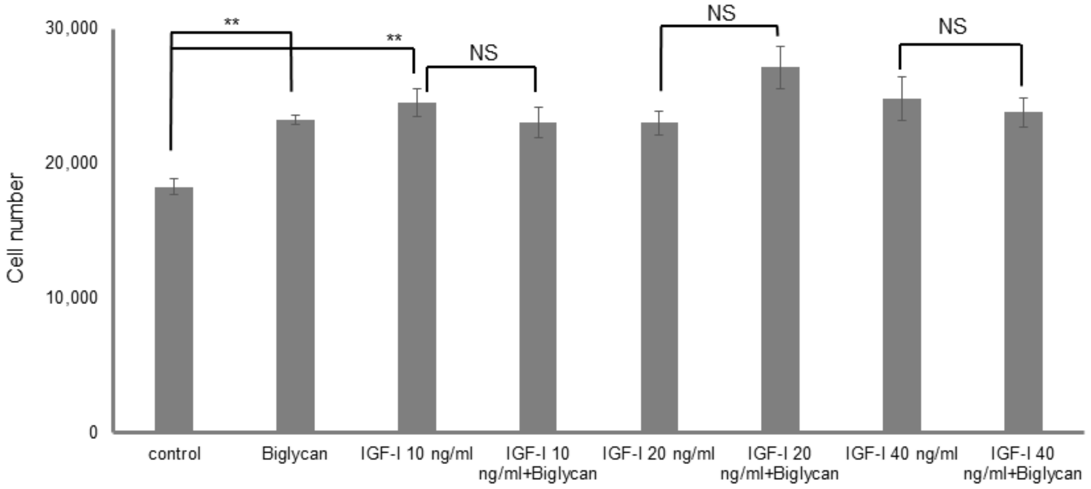

Figure S5. Competition assay between biglycan and IGF-I on MG63 cell proliferation effect. MG63 cells were harvested and seeded on 96-well plates. The next day, cells were treated with a serum-free medium for 24h. MG63 cells were treated with10 μg/mL biglycan, raising concentrations of IGF-I in serum-free culture medium and their combination for 48h. Cells in each well were counted after the incubation period, using a fluorometric CyQUANT assay kit. Results represent the average of three separate experiments. Means ± S.E.M were plotted; statistical significance: **p ≤ 0.01 compared with the respective control samples.

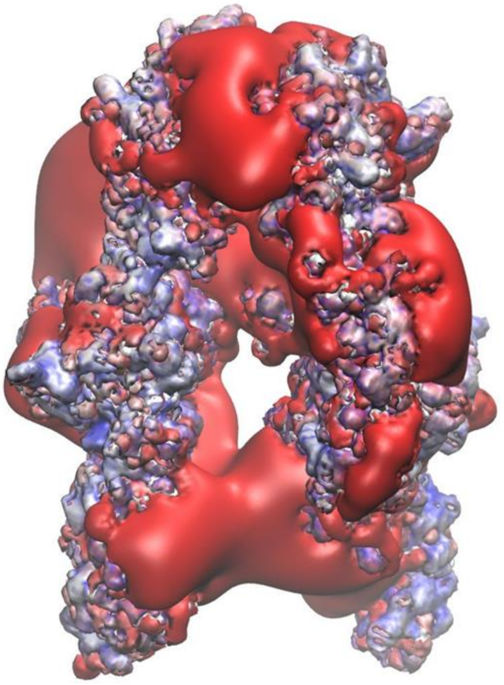

Figure S6. Electrostatic potential in the proximity of the receptor surface. Electrostatic potential isosurfaces were obtained by PBSA calculations for IGF-IR (-2 and 2 kcal/mol/Å in red and blue, respectively).

Supplementary Materials and Methods

Materials

Recombinant human biglycan (AR50812PU-S 1 mg/mL) was obtained from OriGene, Rockville, USA. A specific inhibitor of IGF-IR (AG1024 121767; Calbiochem, San

Diego, USA) was used. Recombinant human IGF-I (insulin-like growth factor I; 291-G1) was obtained from R&D Diagnostics, Minneapolis, USA. Protein A/G PLUS-Agarose (sc-2003) was obtained from Santa Cruz Biotechnology, Inc, Dallas, USA. Primary antibodies from Santa Cruz Biotechnology Inc were used, including anti-biglycan (sc-100857; mouse monoclonal; 1/100 dilution) and anti-GAPDH (sc-47724; mouse monoclonal; 1/200 dilution). The secondary-HRP anti-mouse antibody (AP192P; 1/5.000 dilution) was purchased by EMD Millipore, Burlington, USA.

Cells and Cell Culture

MG63 (ATCC® CRL1427™), Saos-2 (ATCC® HTB-85™), and U-2OS (ATCC® HTB-96™) human osteosarcoma cell lines were utilized. Cells were grown in DMEM (Gibco-41966-029) supplemented with 10% fetal bovine serum (FBS; Invitrogen 10500-064; heat-inactivated), gentamycin (Invitrogen, Waltham, USA; 15710-049) and penicillin/streptomycin (100 units/mL; Biosera LMA4118). Cells were cultured at 37 °C and 5% CO2 conditions.

Proliferation Assay

Growing cells from confluent cultures were seeded in black 96-well plates at a density of 1,500 cells/well (MG63), 4,000 cells/well (U-2OS), and 20,000 cells/well (Saos-2) in 200 μL of DMEM. The cells were allowed to rest overnight, and then the medium was replaced with a serum-free medium. Treatments were added in 0% FBS medium for the next 48 h at 37 °C and 5% CO2. According to the manufacturer's instructions, the cells were then lysed, and their number was calculated using the CyQUANT fluorometric assay (C7026; Thermo Fisher Scientific, Inc.). Fluorescence was measured in a Fluorometer (BioTek/ Agilent Instruments, Inc, Santa Clara, USA.) using the proposed excitation (485 nm) and emission filters (528 nm). A separate standard curve was used to convert fluorescence units to cell numbers. All experiments were performed in triplicate.

Protein Immunoprecipitation

For immunoprecipitation with protein A/G and agarose, after treatments, cells were detached and diluted in 1 mL RIRA solution (50 mMTris-HCl, 1% NP-40, 0.25% Na-Deoxycholate, 150 mM NaCl, 1 mM EDTA with protease and phosphatase inhibitors). One hundred microliters from this dilution were frozen at −80 °C. In the rest, 900 μL of every protein sample, 30 μL of the primary antibody were added, and tubes were incubated on a rotating platform overnight at 4 °C (3 μg of primary antibody for 1 mg of total protein). The next day, 30 μL of protein A/G with agarose (Santa Cruz) were added for 4 h at 4 °C, and tubes were incubated again on a rotating platform. After centrifugation at 1000 rpm for 1 min (4 °C), the precipitate was diluted again with 1 mL RIPA solution. This process was repeated twice more. Finally, the precipitate was diluted in 30 μL 2x dye, and the samples were utilized for Western blot analysis. All the immunoprecipitation experiments were conducted with primary antibodies against proteins whose expression is not affected by

biglycan treatment. The technique of protein immunoprecipitation was optimized, utilizing a known complex as a positive control. Beads without antibody binding or detected with an antibody against GAPDH, which is unrelated to the target protein, were used as negative controls.

Western Blot Analysis

Equal amounts of protein samples were subjected to SDS PAGE using 10% polyacrylamide gels under reducing conditions. Separated protein bands were transferred to nitrocellulose membranes in 10 mM CAPS (pH 11), containing 10% methanol. Membranes were blocked for 1 h at 4 °C with PBS containing 0.1% Tween 20 (PBS Tween) and 5% (w/v) low-fat milk powder. The membranes were incubated overnight at 4 °C on a rotating platform with the primary antibodies in PBS containing 0.1% Tween-20 (PBS-Tween) and 1% (w/v) low-fat milk powder. The immune complexes were detected following incubation with the appropriate peroxidase-conjugated secondary antibody diluted (1:5.000) in PBS-Tween, 2% low-fat milk for 1 h at room temperature, using the LumiSensor Chemiluminescent HRP substrate kit (Genscript; Piscataway, USA L00221V500), according to the manufacturer's instructions.

Statistical Analysis

Statistical significance was evaluated using a Student's t-test, or one-way ANOVA analysis of variance with Tukey's post-test, using GraphPad Prism (version 4.0) software.

2.2.5. MD of Docked Complexes

The structural representatives of the five most populated clusters were prepared in LeaP (AMBER16) [44] from the ClusPro models. The starting structures were subjected to 20 ns of MD-simulation, utilizing a TIP3P octahedral periodic box boundary minimal distance to the solute of 8 Å, and performed as described previously. After simulations, binding free energies and per residue decompositions of the complexes were estimated with the molecular mechanics-generalized Born surface area (MM-GBSA) method in AMBER16. Mode gb = 2 [50] for 200 evenly spaced frames for the 20 ns production run were used for calculations.
