# Supplementary material for: Biglycan Interacts with Type I Insulin-like Receptor (IGF-IR) Signaling Pathway to Regulate Osteosarcoma Cell Growth and Response to Chemotherapy
Source: Cancers (Basel). 2022 Feb 25;14(5):1196. doi: 10.3390/cancers14051196 (PMC8909324; doi:10.3390/cancers14051196)

Figure 1B

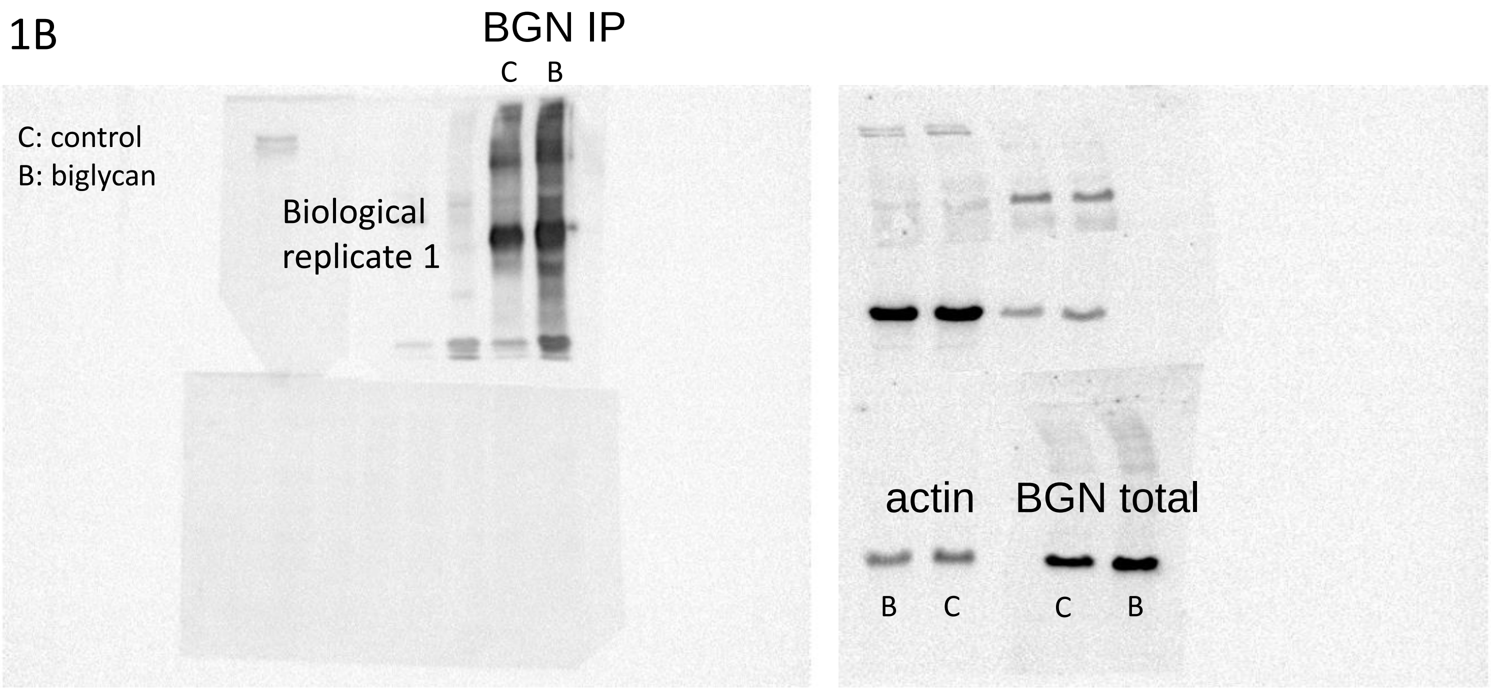
BGN IP
C B
C: control
B: biglycan
Biological
replicate 1
actin
BGN total
B C
C B

Figure 1B

Biological replicates: Biglycan treatments

IP IGF-IR

IB Biglycan

C: control

B: biglycan

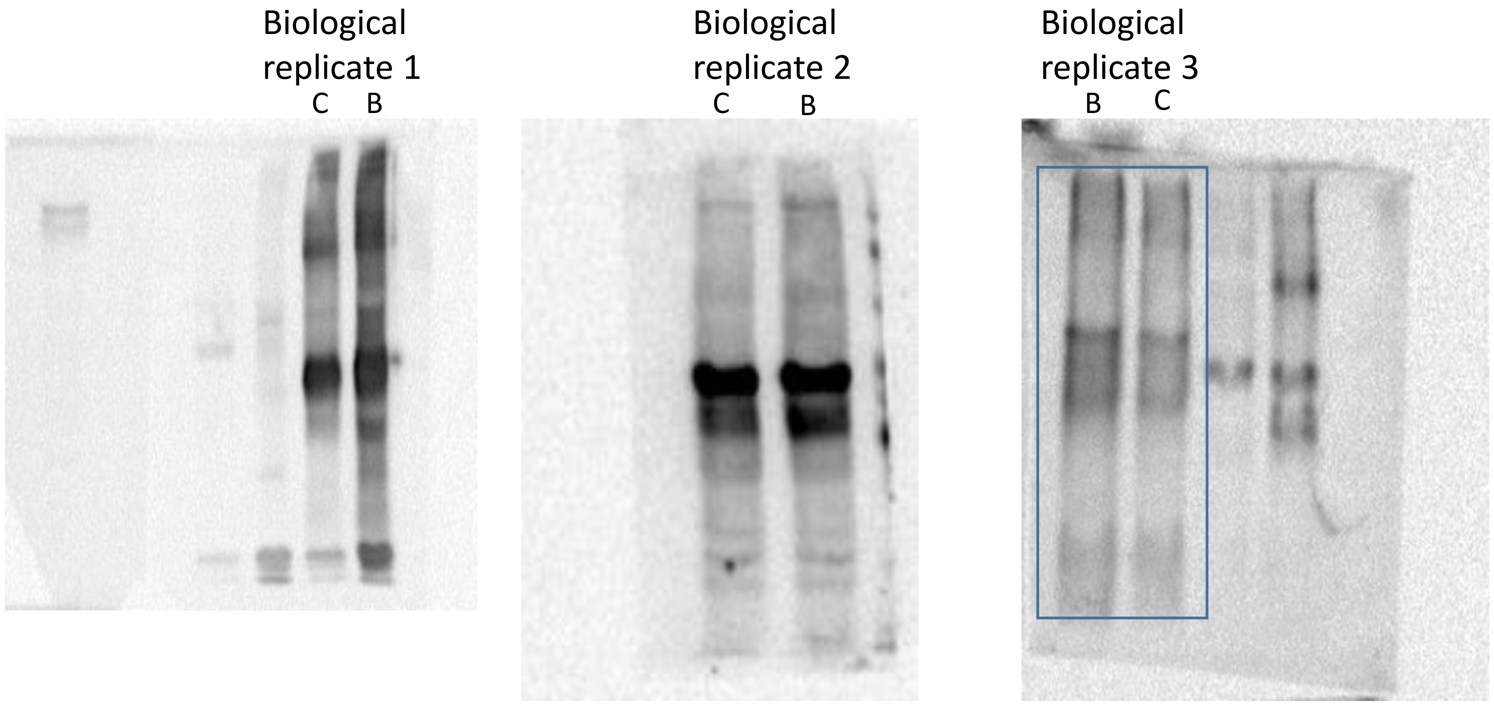

Figure 2A

C: control
B: biglycan

Cyto: cytoplasm
Nucl: nucleus

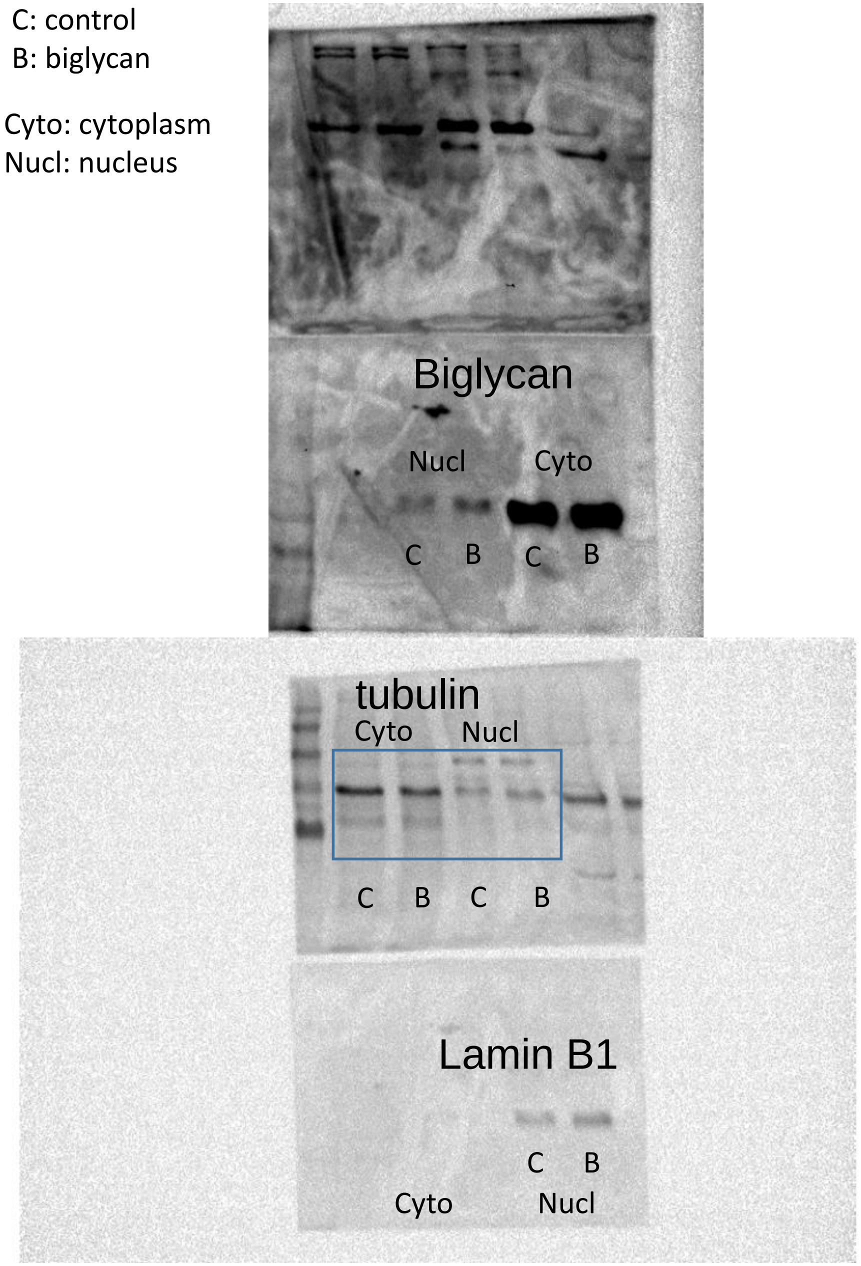

Figure 2C

C: control
B: biglycan

Cyto: cytoplasm
Nucl: nucleus

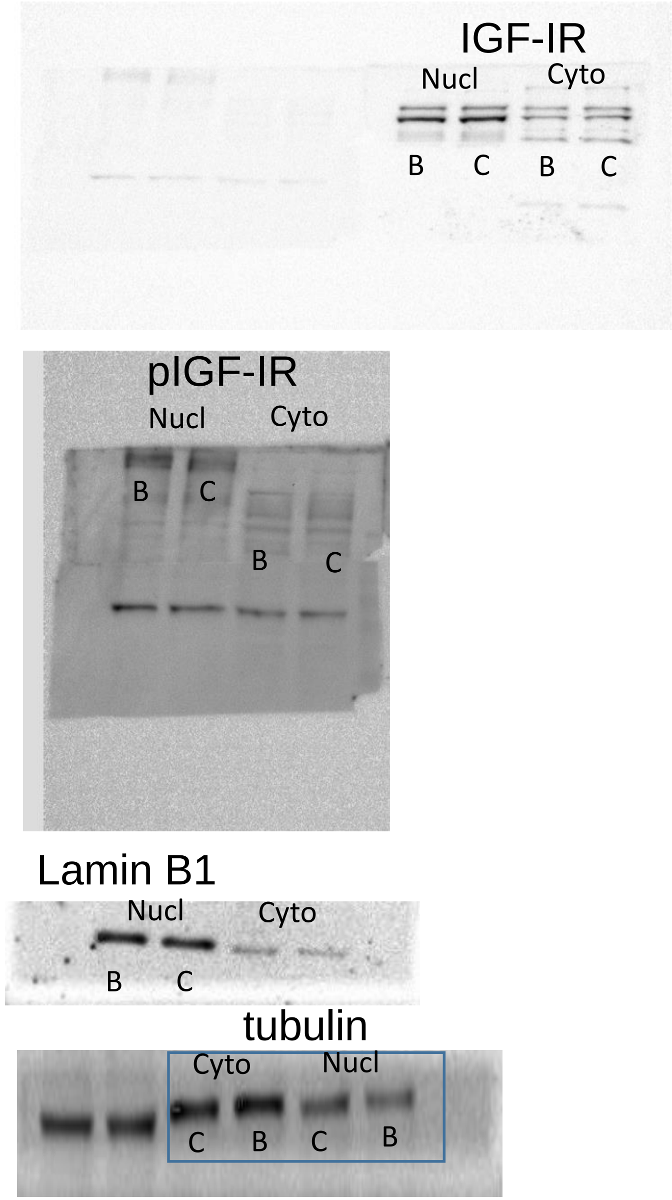

Figure 2A

Biological replicates: Biglycan treatments

Cell fractionation

IB Biglycan

C: control
B: biglycan

Cyto: cytoplasm
Nucl: nucleus

Biological replicate 1

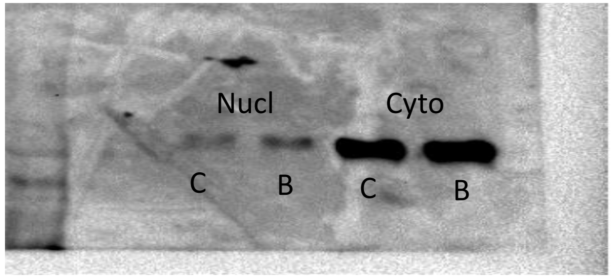

Biological replicate 2

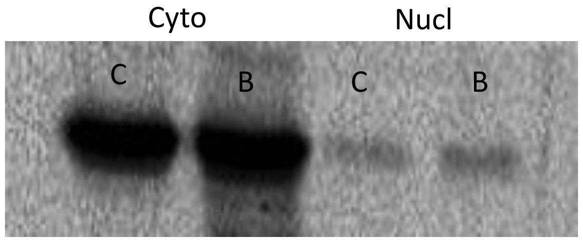

Biological replicate 3

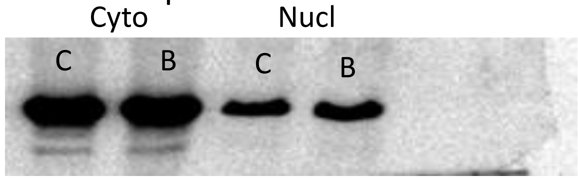

Figure 2C

Biological replicates: Biglycan treatments

Cell fractionation

IB IGF-IR

C: control
B: biglycan

Cyto: cytoplasm
Nucl: nucleus

Biological replicate 1

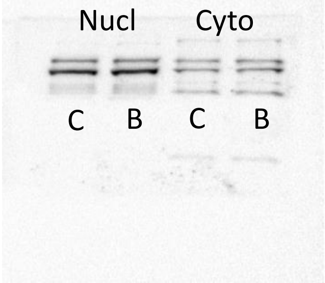

Biological replicate 2

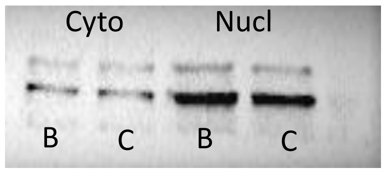

Biological replicate 3

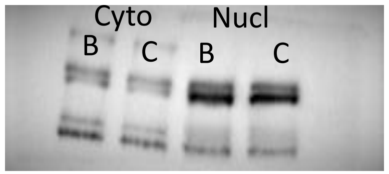

Biological replicates: Biglycan treatments

Cell fractionation

IB pIGF-IR

Biological replicate 1

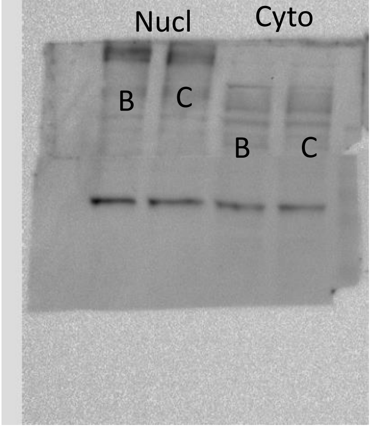

Biological replicate 2

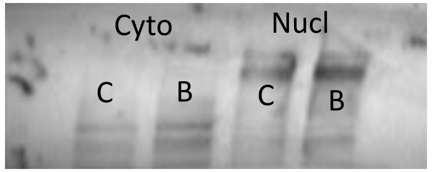

Biological replicate 3

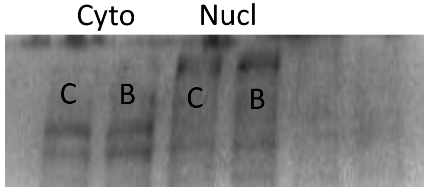

Figure 5A

C: control
B: biglycan

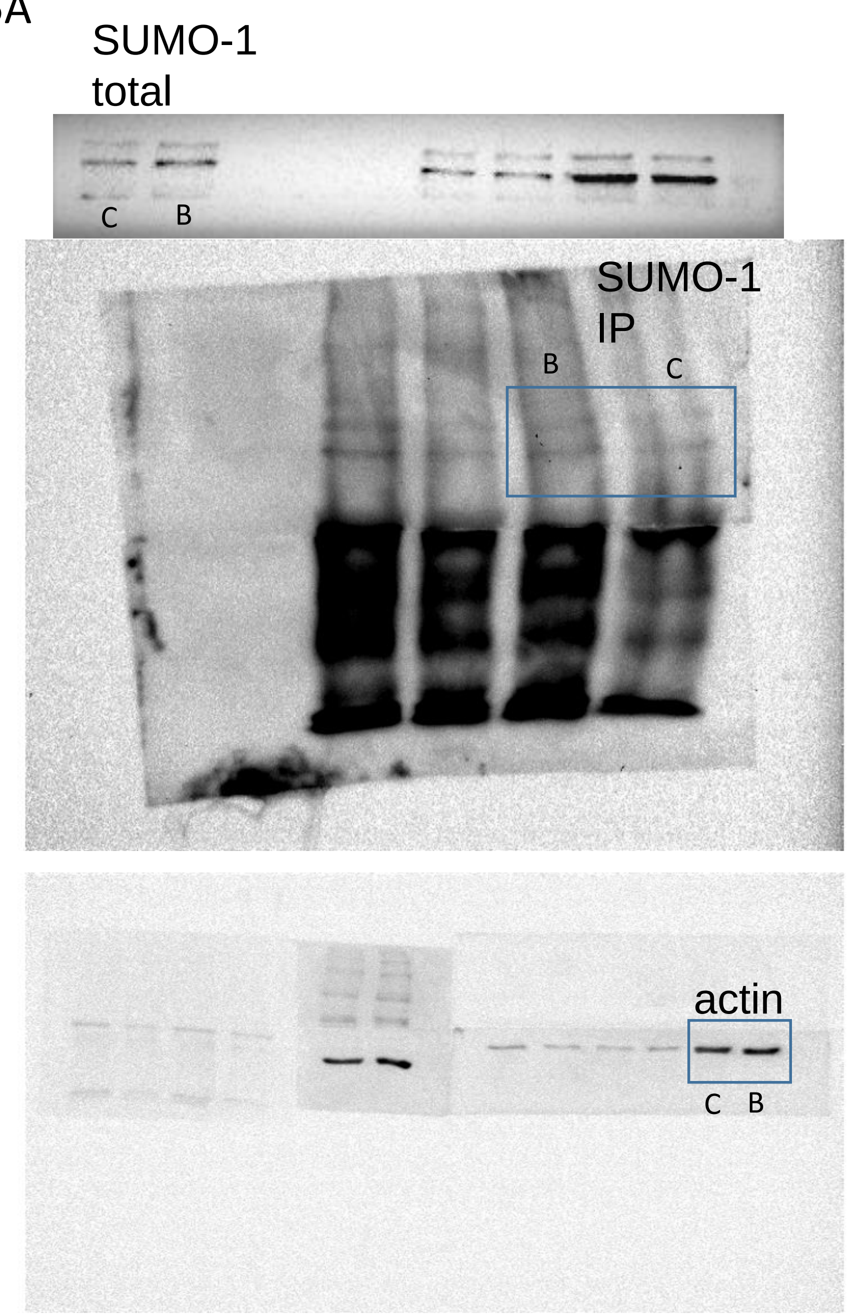

Figure 5C

C: control
B: biglycan

Cyto: cytoplasm
Nucl: nucleus

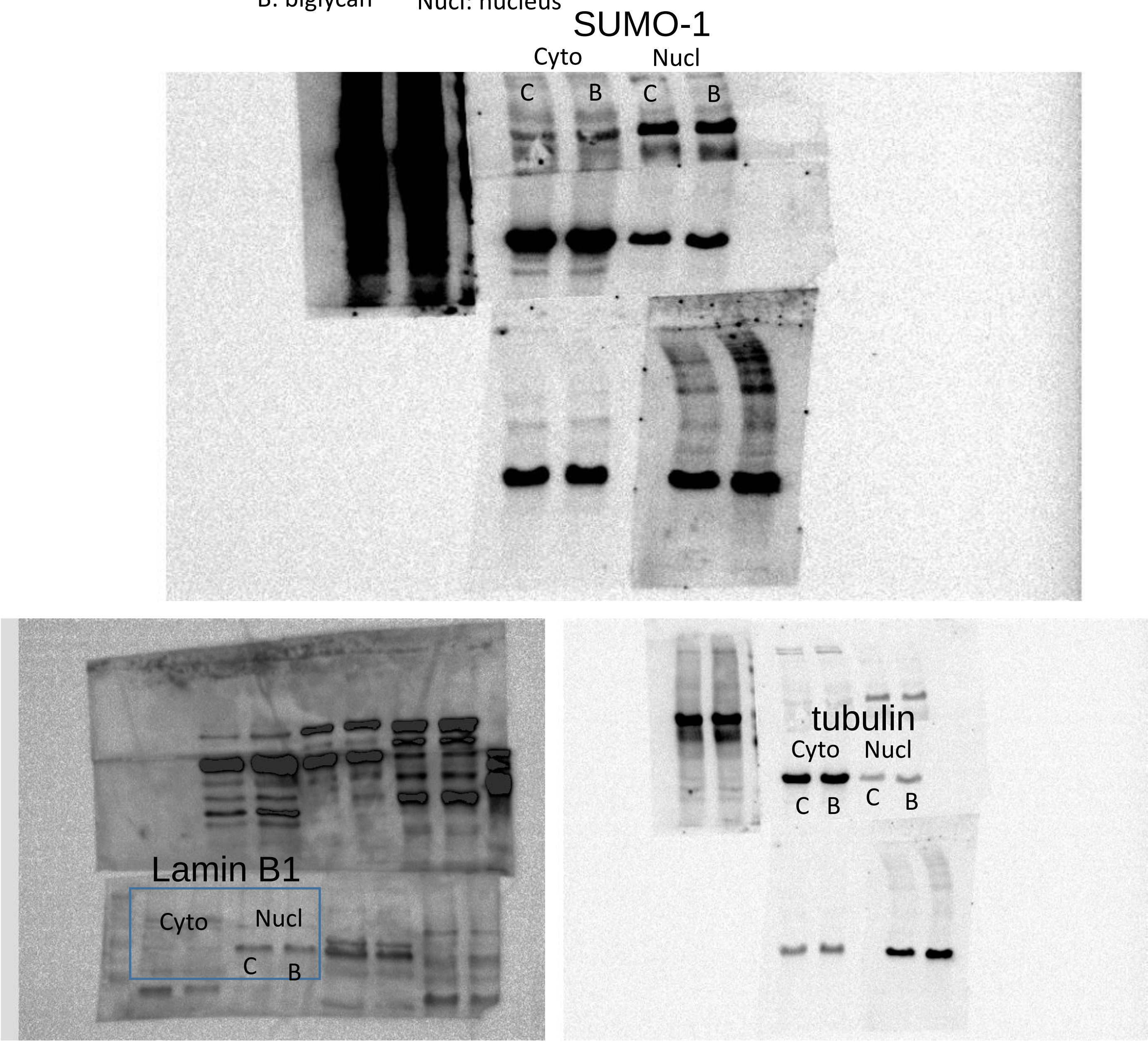

Figure 5A

Biological replicates: Biglycan treatments

IP IGF-IR

IB SUMO-1

C: control
B: biglycan

Biological replicate 1

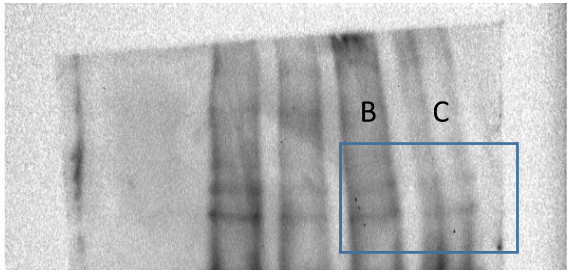

Biological replicate 2

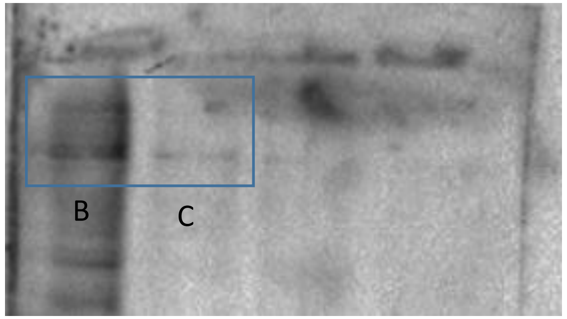

Biological replicate 3

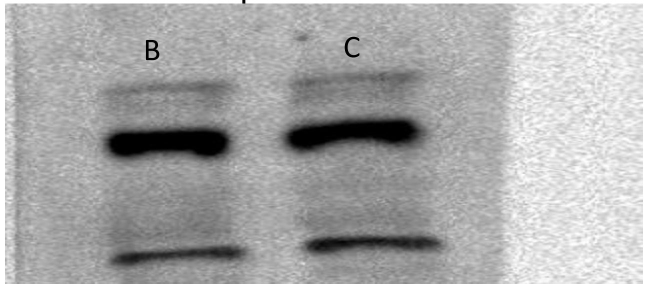

Figure 5C

C: control
B: biglycan

Cyto: cytoplasm
Nucl: nucleus

Biological replicates: Biglycan treatments

Cell fractionation

IB SUMO-1

Biological replicate 1

Cyto Nucl

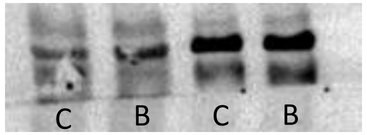

Biological replicate 2

Cyto Nucl

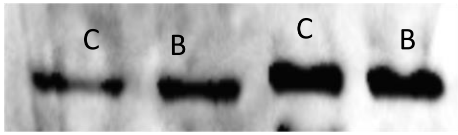

Biological replicate 3

Cyto Nucl

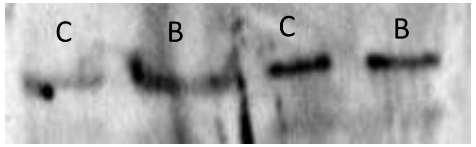

Figure 7A D: DMSO Cyto: cytoplasm
A: AG1024 Nucl: nucleus

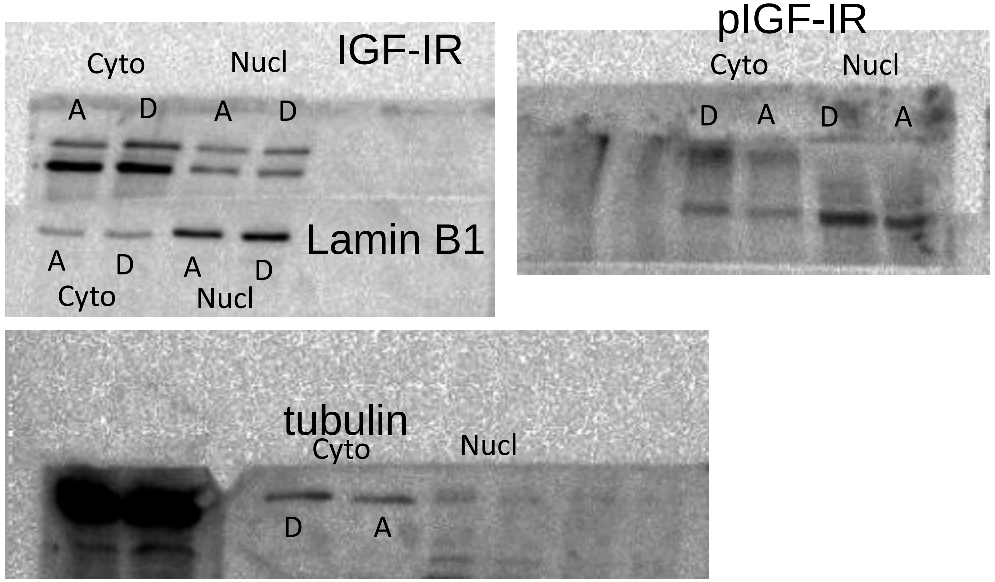

Figure 7C D: DMSO Cyto: cytoplasm
A: AG1024 Nucl: nucleus

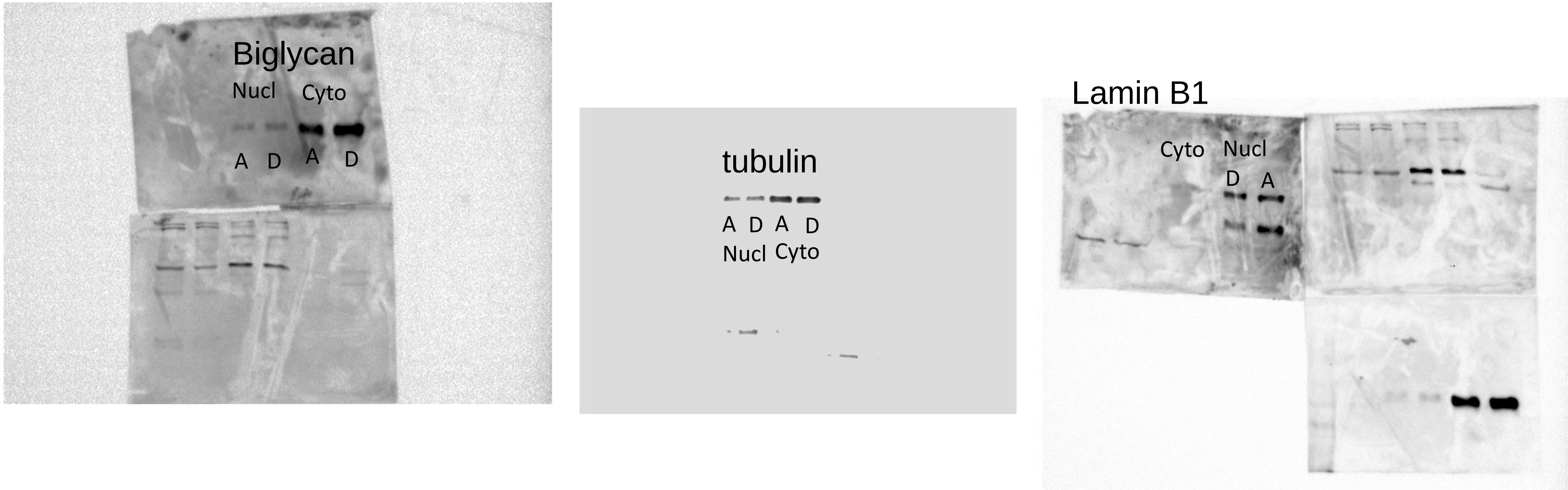

Figure 7A

D: DMSO
A: AG1024

Cyto: cytoplasm
Nucl: nucleus

Biological replicates: AG1024 treatments
Cell fractionation
IB IGF-IR

Biological replicate 1

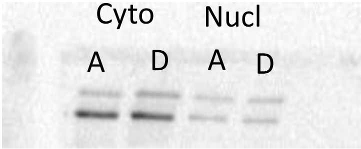

Biological replicate 2

Cyto Nucl

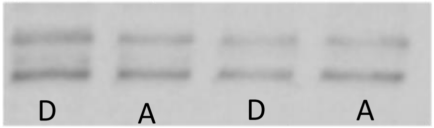

Biological replicate 3

Cyto Nucl

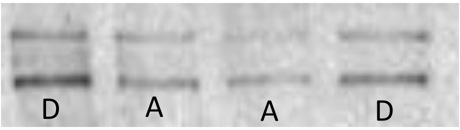

Biological replicates: Biglycan treatments
Cell fractionation
IB pIGF-IR

Biological replicate 1

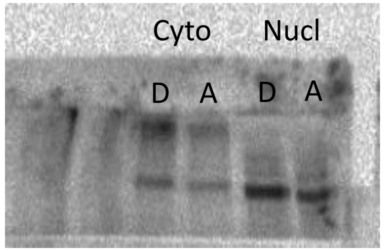

Biological replicate 2

Cyto Nucl

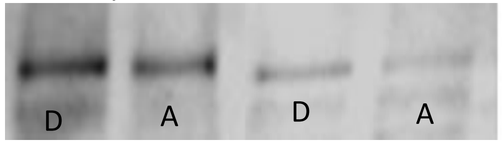

Biological replicate 3

Cyto Nucl

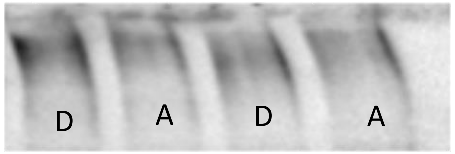

Figure 7C

D: DMSO
A: AG1024

Cyto: cytoplasm
Nucl: nucleus

Biological replicates: AG1024 treatments
Cell fractionation
IB Biglycan

Biological replicate 1

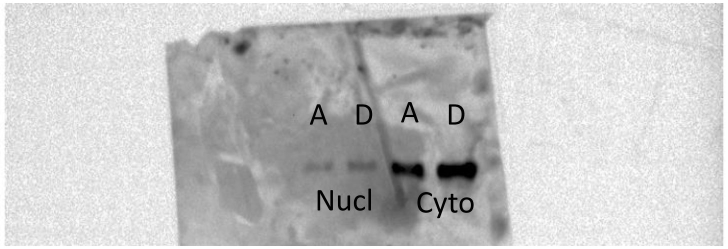

Biological replicate 2

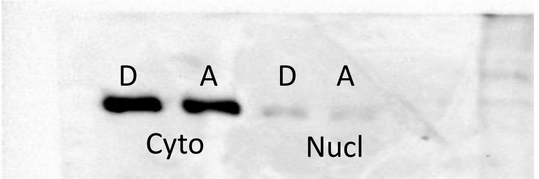

Biological replicate 3

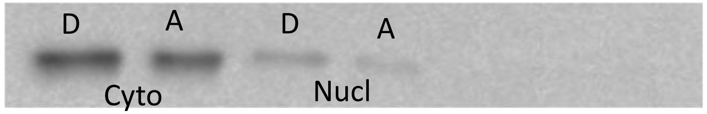

Figure 7E

D: DMSO
A: AG1024

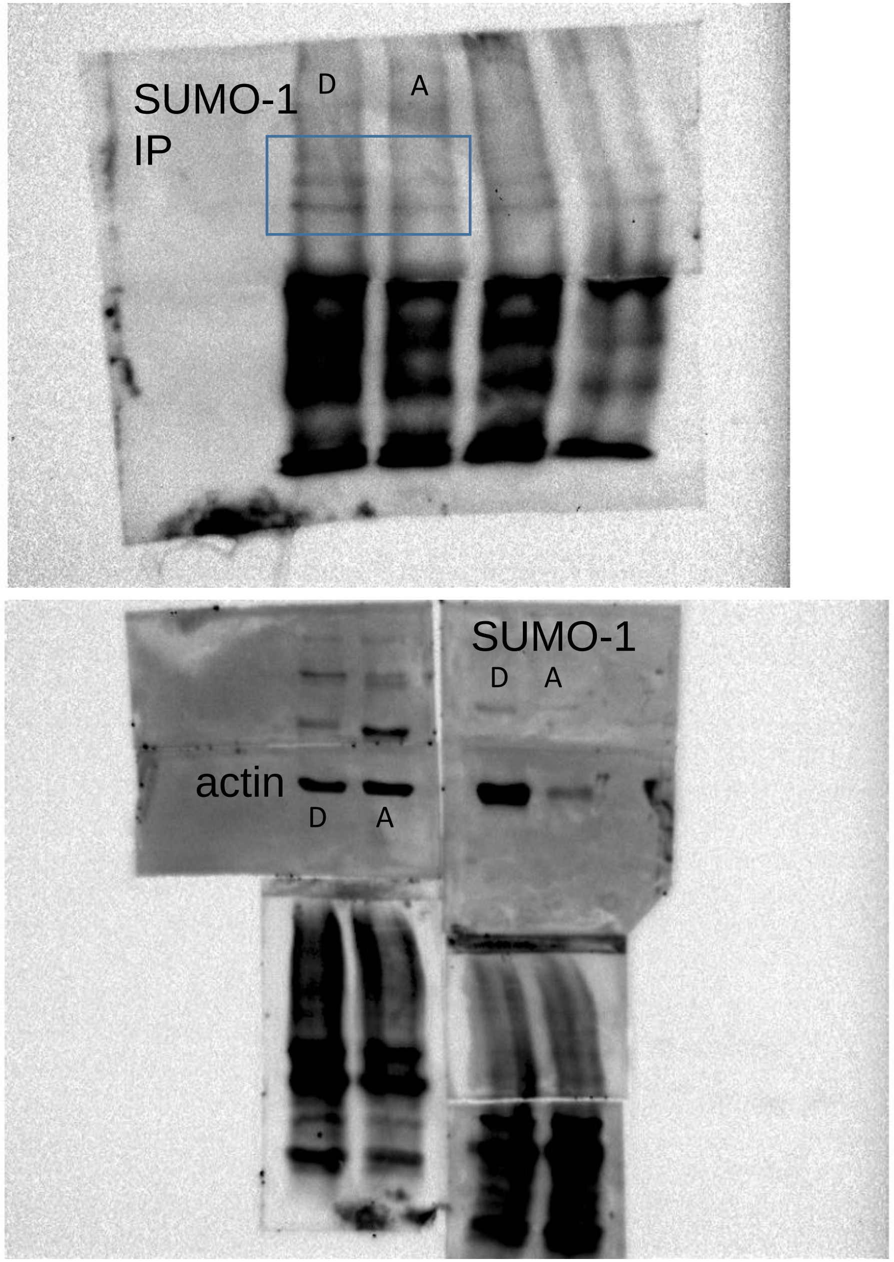

Figure 7G

D: DMSO
A: AG1024
Cyto: cytoplasm
Nucl: nucleus

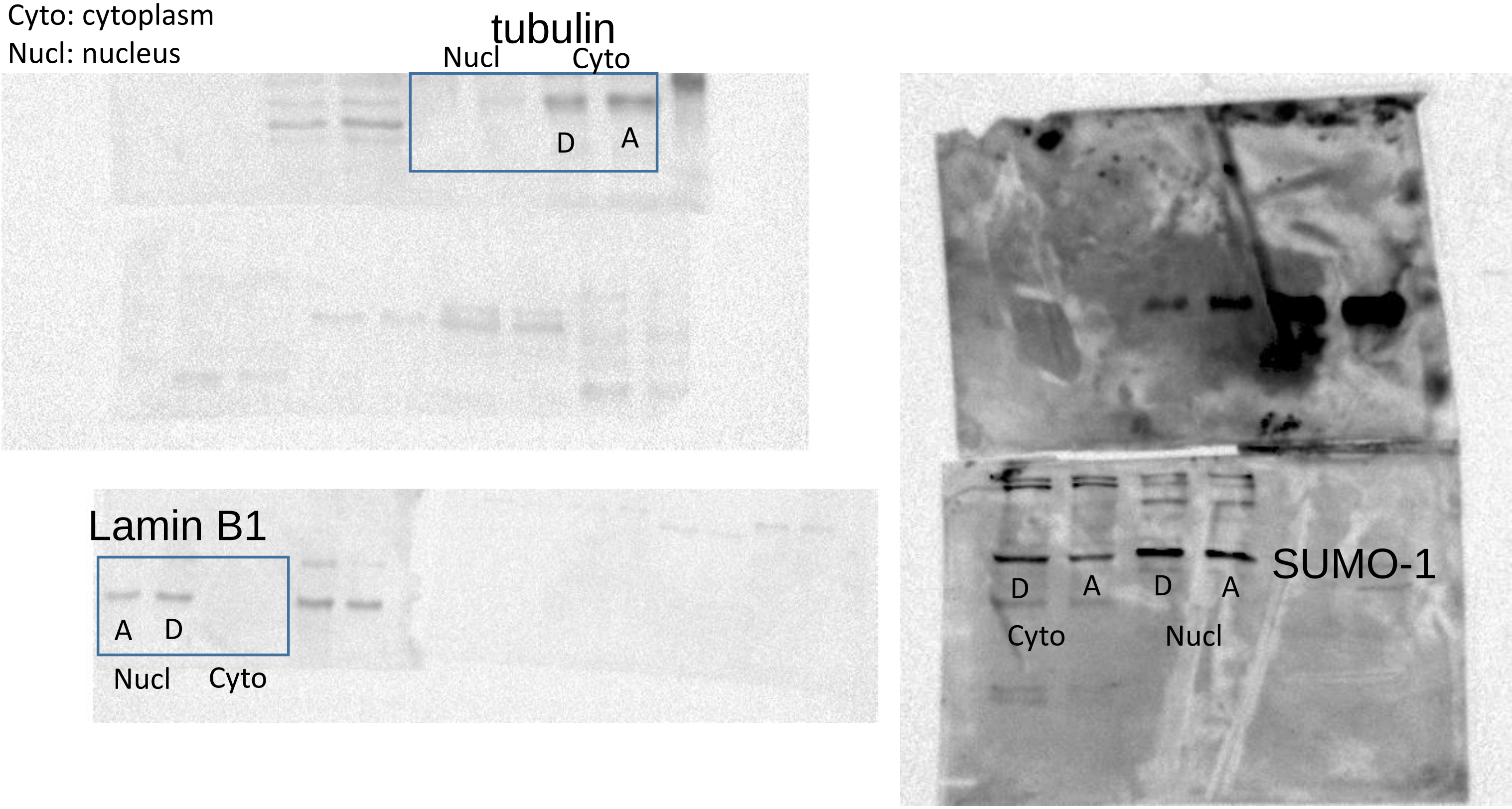

Figure 7E

Biological replicates: AG1024 treatments

IP IGF-IR

IB SUMO-1

D: DMSO

A: AG1024

Biological replicate 1

Biological replicate 2

Biological replicate 3

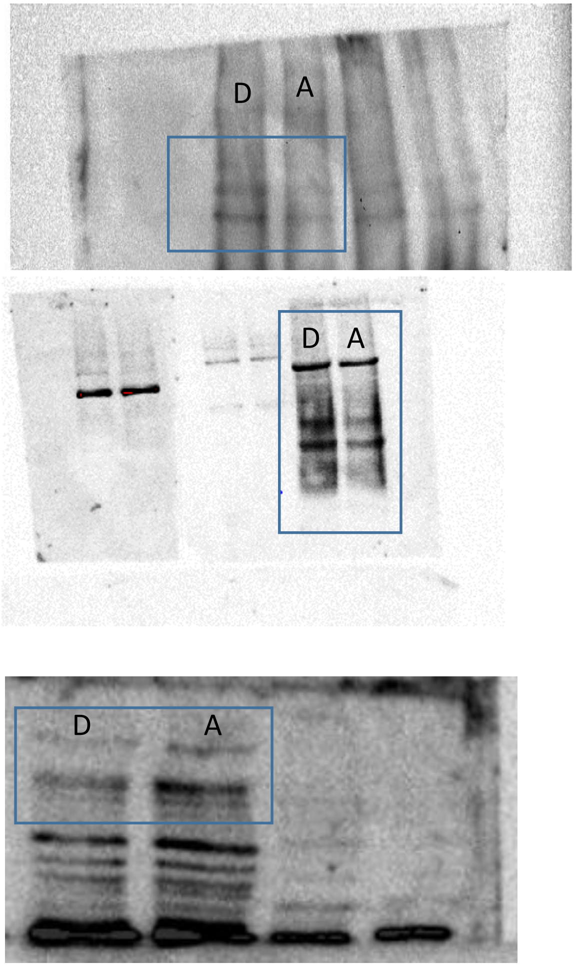

Figure 7G

Biological replicates: AG1024 treatments

Cell fractionation

IB SUMO-1

D: DMSO
A: AG1024

Cyto: cytoplasm
Nucl: nucleus

Biological replicate 1

Biological replicate 2

Biological replicate 3

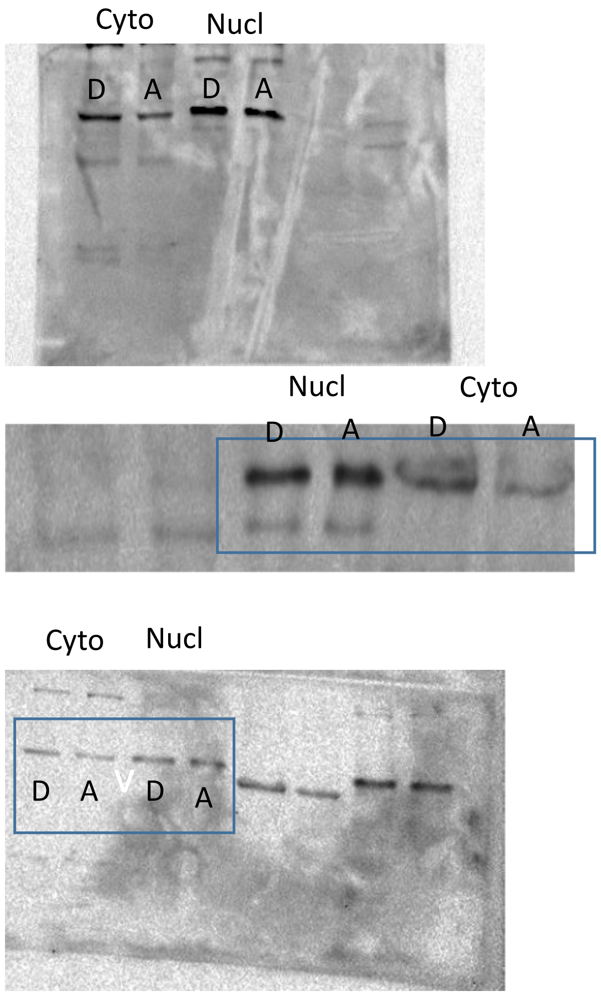

Figure 8B

C: control

B: biglycan

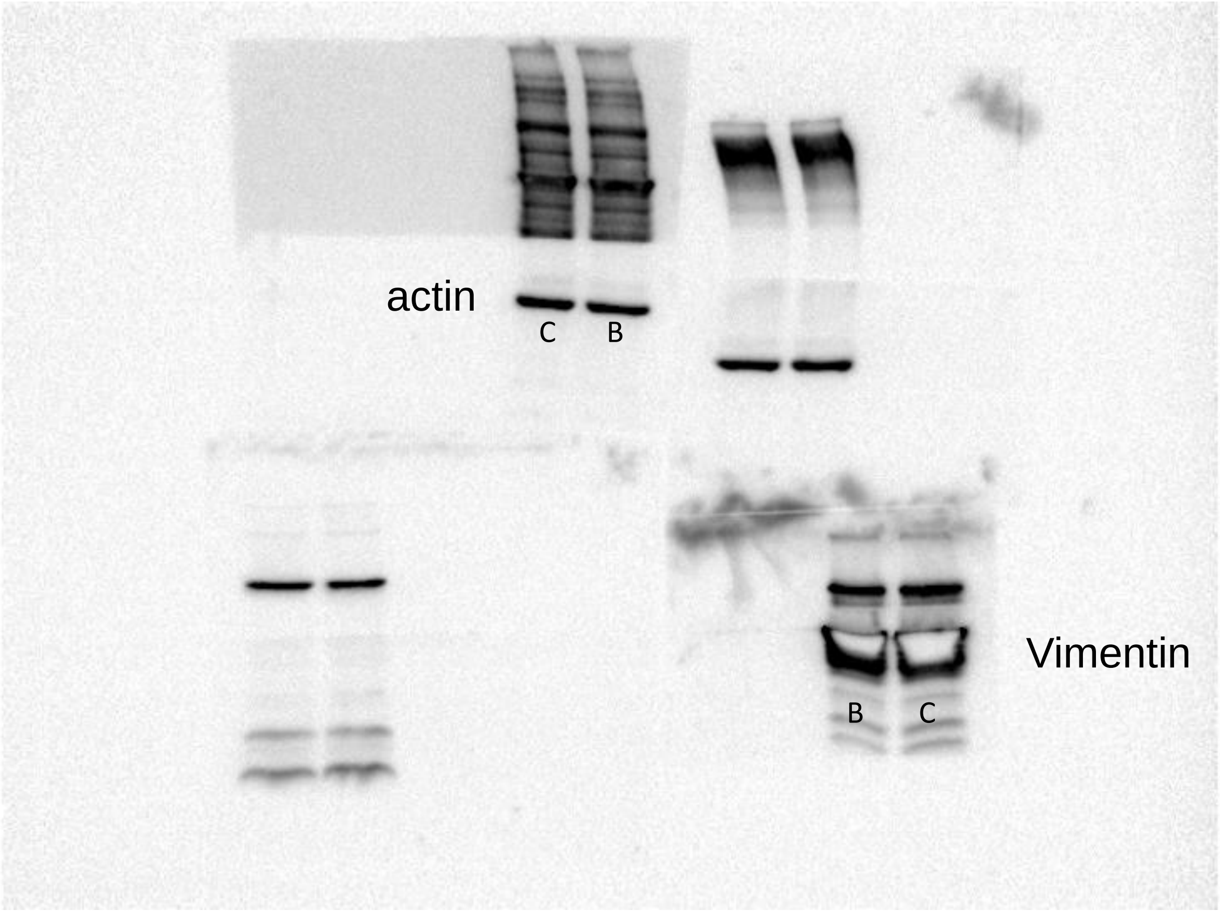

Figure 8D

C: control

B: biglycan

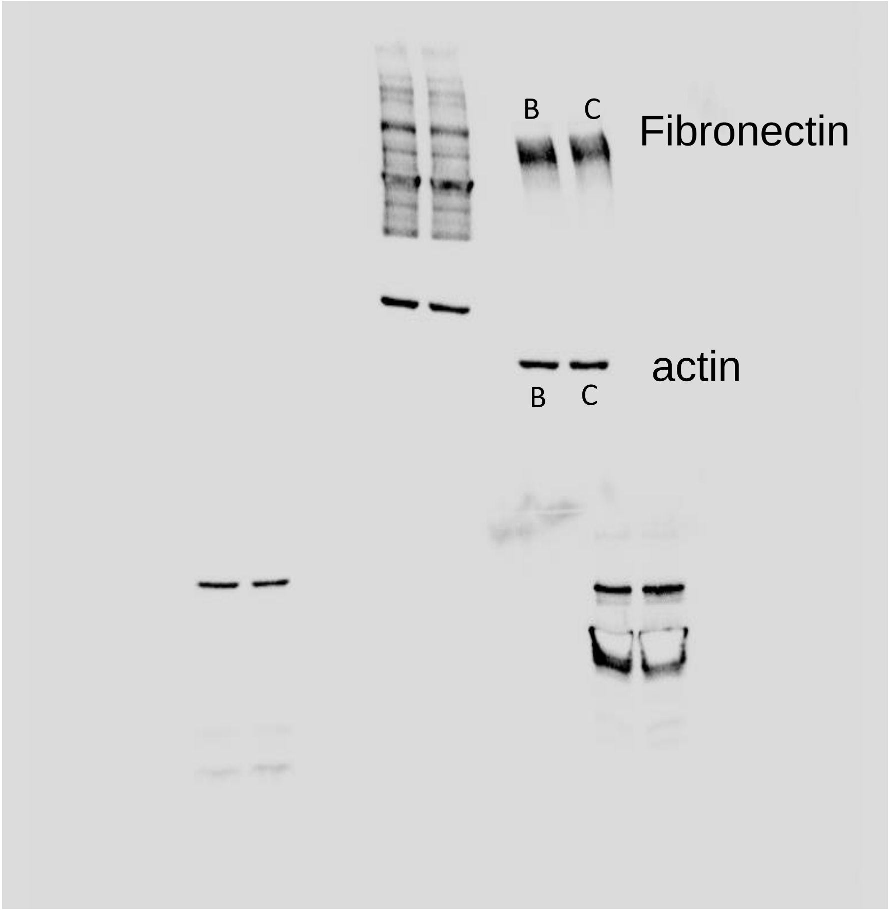

Figure 8B

Biological replicates: Biglycan treatments

IB Vimentin

C: control

B: biglycan

Biological replicate 1 | Biological replicate 2

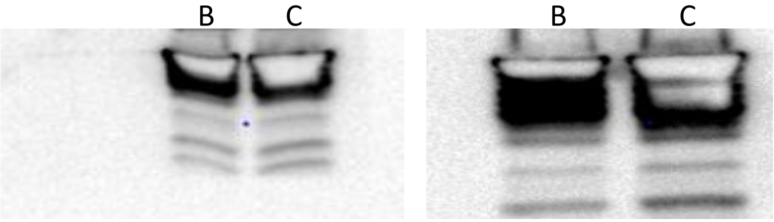

Figure 8D

Biological replicates: Biglycan treatments

IB Fibronectin

C: control

B: biglycan

Biological replicate 1 | Biological replicate 2

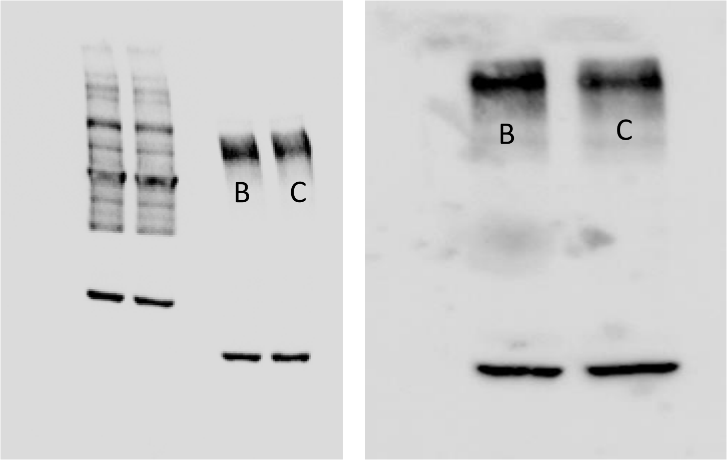

Supplement: Supplementary file 1 [file cancers-14-01196-s001.zip › cancers-1562188.WB FIGURESpdf.pdf]
